# Supplementary material for: Genome-wide association and Mendelian randomisation analysis among 30,699 Chinese pregnant women identifies novel genetic and molecular risk factors for gestational diabetes and glycaemic traits
Source: Diabetologia. 2024 Feb 19;67(4):703–13. doi: 10.1007/s00125-023-06065-5 (PMC10904416; doi:10.1007/s00125-023-06065-5)
Supplement: Supplementary file 1 — Supplementary file1 (PDF 7.51 MB) [file 125_2023_6065_MOESM1_ESM.pdf]

Electronic supplementary material(ESM) for

Genome-wide association and Mendelian randomisation analysis among 30,699 Chinese pregnant women identifies novel genetic and molecular risk factors for gestational diabetes and glycaemic traits

Jianxin Zhen[1,2#], Yuqin Gu[2#], Piao Wang[2], Weihong Wang[1], Shengzhe Bian[2], Shujia Huang[3], Hui Liang[1,4], Mingxi Huang[3], Yan Yu[5], Qing Chen[6], Guozhi Jiang[2], Xiu Qiu[3,7], Likuan Xiong[1,4*], Siyang Liu[2*]

[1]Central Laboratory, Shenzhen Baoan Women's and Children's Hospital, Shenzhen, Guangdong, China
[2]School of Public Health (Shenzhen), Shenzhen Campus of Sun Yat-sen University, Shenzhen, Guangdong, China
[3]Division of Birth Cohort Study, Guangzhou Women and Children's Medical Center, Guangzhou Medical University, Guangzhou, China
[4]Shenzhen Key Laboratory of Birth Defects Research, Shenzhen, Guangdong, China
[5]Department of Obstetrics, Shenzhen Baoan Women's and Children's Hospital, Shenzhen, Guangdong, China
[6]Department of Pharmacy, Shenzhen Baoan Women's and Children's Hospital, Shenzhen, Guangdong, China
[7]Department of Women's Health, Provincial Key Clinical Specialty of Woman and Child Health, Guangzhou Women and Children's Medical Center, Guangzhou Medical University, Guangzhou, China

Jianxin Zhen and Yuqin Gu contributed equally to this study

Corresponding authors:
Siyang Liu, liusy99@mail.sysu.edu.cn
Likuan Xiong, xionglk@sina.cn

Received: 11 August 2023 / Accepted: 3 November 2023

Table of contents

ESM Methods *4*

The NIPT PLUS cohort 4

Statistical test of different genetic effects between two GWAS........ 4

Supplementary Tables *5*

ESM Table 1 Geographic distribution of the participants in this study. 5

ESM Table 2 55 biomarkers list that included in this study 7

ESM Table 3 Clinical characteristics of the study participants........ 8

ESM Table 4 Statistical test results for clinical characteristics........ 9

ESM Table 5 Heritability of GDM and related quantitative trait and the genetic correlation with T2D. 10

ESM Table 6 Heritability of FPG and OGTT2H in the MAGIC consortium. 12

ESM Table 7 Replication of the lead SNPs in the NIPT PLUS, BIGCS cohort and meta-analysis........ 13

ESM Table 8 The information of Lead SNPs about the five traits investigated in the study. 15

ESM Table 9 Comparison of the 13 significnat loci for FPG and OGTT2H in the MAGIC consortium East Asian population. 17

ESM Table 10 Detection specific SNP genetic effects of FPG lead SNPs with OGTT1H and OGTT2H . 18

ESM Table 11 MR results of GDM and 4 quantitative glycemic traits and biomarkers........ 19

ESM Table 12 Instrumental variables of biomarkers as exposure and GDM as outcome........ 20

ESM Table 13 The power of Mendelian Randomization(MR) analyses........ 23

ESM Table 14 MR results of GDM and 4 quantitative glycemic traits and 4 biomarkers use meta data with PLUS cohort. 24

Supplementary Figures *28*

ESM Fig. 1 Multiple regression analyses of GDM and the four glycemic traits........ 28

ESM Fig. 2 Power analysis of the genome-wide association analysis. 29

ESM Fig. 3 Manhattan plot of GDM case with medication or not (case/control=419/2898). 30

ESM Fig. 4 Comparison of the genome-wide association analysis for FPG and OGTT2H between MAGIC East Asian and our study. 31

ESM Fig. 5 Manhattan and qq plot of OGTT1H(a) and OGTT2H(b) with age,BMI, gestational week of OGTT and FPG as covariants........ 32

ESM Fig. 6 Locuszoom plot of genome-wide significant loci associated with the seven traits investigated in the study........ 38

ESM Fig. 7 Effects of biomarkers on GDM and four glycemic traits by mendelian randomization.... 39

ESM Fig. 8 Effects of all 51 biomarkers on GDM and glycemic traits by mendelian randomization.. 40

ESM Fig. 9 Effects of GDM and glycemic traits on the biomarkers by Mendelian randomization..... 41

ESM Fig. 10 Effects of GDM and glycemic traits on all the 55 biomarkers by Mendelian randomization. 42

ESM Fig. 11 Scatter plot of mendelian randomization analysis results with 4 biomarkers as the exposure and their effect on GDM with PLUS cohort meta data. 43

ESM Methods

The NIPT PLUS cohort

In addition to the previously mentioned participants, we collected data from 4,688 individuals who sought maternity check-ups at Shenzhen Baoan Women's and Children's Hospital (Shenzhen, China) throughout the entire 40-week gestational period. These individuals underwent NIPT in either the first or second trimester between 2020 and 2021. Notably, these samples are characterized by a deeper sequencing depth in comparison to conventional NIPT, with an average depth of 0.3x. For this study, we leveraged their recorded data on physical measurement, blood glucose levels, lymphocyte percentage, white blood cell count, neutrophil percentage, absolute neutrophil count, and clinical diagnostic information related to GDM during pregnancy.

Statistical test of different genetic effects between two GWAS

In this study, we compared the GWAS results with those from MAGIC and between the baseline glucose and the challenged glucose levels. We examined the difference in genetic effects between two GWAS with a two-sided *two-sample t-test* with the following hypotheses:

$$Null\ hypothesis\ H_0: \beta_1 = \beta_2$$
$$Alternative\ hypothesis\ H_1: \beta_1 \neq \beta_2$$

The T statistic was computed as follows:

$$T = \frac{\beta_1 - \beta_2}{\sqrt{\frac{S_1^2}{n_1} + \frac{S_2^2}{n_2}}} = \frac{\beta_1 - \beta_2}{\sqrt{SE_1^2 + SE_2^2}} \sim t(v') \quad (1)$$

The degrees of freedom v' was determined by the formula:

$$v' = \frac{(SE_1^2 + SE_2^2)^2}{\frac{SE_1^4}{n_1 - 1} + \frac{SE_2^4}{n_2 - 1}} \quad (2)$$

Herein, β_1 and β_2 represent the genetic effects associated with specific traits (for example, baseline glucose levels) and other traits (for example, challgend glucose levels), respectively. S_1^2 and S_2^2 denote sample variance, whereas SE_1 and SE_2 stand for estimated standard errors. It is established that the T statistic in equation (1) follows a t-distribution with a degree of freedom v'. To address potential inequality between S_1^2 and S_2^2, the adjusted v' was employed, computed according to formula (2).

For all the GWAS loci we conducted, we used the P-value threshold ($P < 0.05$) to define statistical significance and reported the specific genetic locus.

Supplementary Tables

ESM Table 1 Geographic distribution of the participants in this study.

Province	Administrative Definiation of Region	Samplesize
Guangdong	South	11161
Hunan	South	4911
Hubei	Central	3337
Jiangxi	South	2684
Guangxi	South	1909
Henan	Central	1303
Sichuan	Central	1193
Anhui	Central	511
Shaanxi	North	461
Fujian	South	450
Shandong	North	325
Heilongjiang	North	319
Guizhou	South	287
Chongqing	Central	286
Jilin	North	195
Hebei	North	170
Liaoning	North	161
Hainan	South	160
Gansu	North	154
Jiangsu	Central	152
Shanxi	North	130
Yunnan	South	122
Zhejiang	Central	114
Neimenggu	North	83
Xinjiang	North	38

Ningxia	North	17
Qinghai	North	14
Tianjin	North	13
Beijing	North	3
Shanghai	Central	2
Tibet	Central	1

ESM Table 2 55 biomarkers list that included in this study

Category	Biomarkers	Category	Biomarkers
Blood routine (N=28)	Absolute_neutrophils	Blood anemia (N=6)	25_hydroxyvitamin_D_D2+D3
	Absolute_value_of_basophils		Ferritin
	Basophil_percentage		Folic_acid
	Eosinophil_absolute_value		HemoglobinA2
	Eosinophil_percentage		HemoglobinF
	Hematocrit		Vitamin_B12
	Hemoglobin_concentration	Blood lipid (N=2)	Total_cholesterolt
	Immature_granulocyte_percentage		Triglyceride
	Immature_granulocytes	Kidney function (N=3)	Creatinine
	Large_platelet_ratio		Urea
	Lymphocyte_absolute_value		Uric_acid
	Lymphocyte_percentage	Liver function (N=8)	Alanine_transaminase
	Mean_platelet_volume		Albumin
	Mean_red_blood_cell_hemoglobin_concentration		Aspartate_aminotransferase
	Mean_red_blood_cell_hemoglobin_content		Globulin
	Mean_red_blood_cell_volume		Total_bile_acid
	Monocyte_absolute_value		Total_bilirubin
	Monocyte_percentage		Total_protein
	Neutrophil_percentage		White_ball_ratio
	Nucleated_red_blood_cell_absolute_value	Thyroid function (N=3)	Free_thyroxine
	Nucleated_red_blood_cells		Nntithyroid_peroxidase_antibody
	Platelet_count		Thyroid_stimulating_hormone
	Platelet_hematocrit	Infection (N=5)	Cytomegalovirus_IgM_antibody_quantitative
	Platelet_volume_distribution_width		Herpes_simplex_virus_typeI_IgM_antibody_quantitative
	Red_blood_cell_count		Herpes_simplex_virus_typeII_IgM_antibody_quantitative
	Red_blood_cell_volume_distribution_width_variation		Rubella_virus_IgM_antibody_quantitative
	Red_blood_cell_volume_distribution_width		Toxoplasma_IgM_antibody_quantitative
	White_blood_cell		

ESM Table 3 Clinical characteristics of the study participants

	GDM	Nondiabetic control
**N**	3,317	19,565
**Age (years)**	31.50±4.23	29.32±3.98
**Weight(kg)**	57.02±8.73	54.18±7.59
**BMI (kg/m^2)**	22.74±3.21	21.45±2.76
**SBP (mmHg)**	116.73±13.67	114.66±12.95
**DBP (mmHg)**	68.71±10.30	66.84±9.95
**FPG (mmol/L)**	4.61±0.51	4.34±0.32
**OGTT0H (mmol/L)**	4.80±0.51	4.36±0.31
**OGTT1H (mmol/L)**	9.84±1.39	7.28±1.31
**OGTT2H (mmol/L)**	8.59±1.33	6.36±1.00

Shown is the mean and the standard deviation of the measurements. N refers to sample size. BMI: Body mass index; SBP: Systolic blood pressure; DBP: Diastolic blood pressure; FPG: Fasting plasma glucose; OGTT0H, 1H and 2H: Oral glucose tolerance test 0 hour, 1 hour and 2 hours.

ESM Table 4 Statistical test results for clinical characteristics.

	GDM		FPG		OGTT0H		0GTT1H		OGTT2H	
	**OR**	***P***	**beta**	***P***	**beta**	***P***	**beta**	***P***	**beta**	***P***
Age (years)	1.13	<0.0001	0.03	<0.0001	0.04	<0.0001	0.06	<0.0001	0.06	<0.0001
Weight(kg)	1.42	<0.0001	0.20	<0.0001	0.23	<0.0001	0.15	<0.0001	0.13	<0.0001
BMI (kg/m^2)	1.56	<0.0001	0.20	<0.001	0.24	<0.0001	0.20	<0.0001	0.19	<0.0001
SBP (mmHg)	1.01	<0.0001	0.002	<0.0001	0.005	<0.0001	0.005	<0.0001	0.004	<0.0001
DBP (mmHg)	1.01	<0.0001	0.002	<0.0001	0.005	<0.0001	0.005	<0.0001	0.004	<0.0001

The qualitative and quantitative variables were analyzed by logictic regression and linear regression analysis without covariate, respectively.GDM: Gestational diabetes mellitus; PGDM: Pre-gestational diabetes mellitus; BMI: Body mass index; SBP: Systolic blood pressure; DBP: Diastolic blood pressure; FPG: Fasting plasma glucose; OGTT0H, 1H and 2H: Oral glucose tolerance test 0 hour, 1 hour and 2 hours.

ESM Table 5 Heritability of GDM and related quantitative trait and the genetic correlation with T2D.

	GDM	FPG	OGTT0H	OGTT1H	OGTT2H
Sample size	3317:19565	26751	24929	24931	24931
Total Observe scale $\boldsymbol{h_g^2}$	0.032(0.019)	0.053(0.019)	0.096(0.027)	0.102(0.022)	0.078(0.020)
Lambda GC	1.032	1.023	1.038	1.068	1.056
Mean Chi^2	1.035	1.036	1.074	1.086	1.069
Intercept	1.020(0.006)	1.007(0.007)	1.024(0.008)	1.032(0.008)	1.027(0.008)
Ratio	0.576(0.176)	0.178(0.179)	0.324(0.103)	0.368(0.091)	0.390(0.118)
Genetic correlation^{AF}	0.842(0.305)	0.456(0.188)	0.523(0.112)	0.737(0.105)	0.730(0.118)
P^{AF}	0.006	0.015	2.77E-06	2.71E-12	6.02E-10
Genetic correlation^{A}	0.806(0.240)	0.307(0.136)	0.196(0.065)	0.619(0.079)	0.695(0.100)
P^{A}	8.0E-04	0.024	0.003	3.94E-15	3.88E-12
Genetic correlation^{E}	0.509(0.205)	0.145(0.078)	0.458(0.093)	0.307(0.064)	0.352(0.073)
P^{E}	0.013	0.062	7.75E-07	1.37E-06	1.28E-06

GDM: Gestational diabetes mellitus; FPG: Fasting plasma glucose; OGTT0H, 1H and 2H: Oral glucose tolerance test 0 hour, 1 hour and 2 hours.

[A]:East Asian T2D; [AF]:Asian femaleT2D; [E]:European T2D; Total Observe scale Asian T2D h_g^2: 0.078(0.0074); Total Observe scale Asian female T2D h_g^2: 0.0664(0.0067);Total Observe scale European T2D h_g^2: 0.0692(0.0097). East Asian T2D: the data from the East Asian meta-analyses of 23 T2D GWAS in 433,540 individuals from China, Japan, Korea, Philippines and Malaysia participating in the Asian Genetic Epidemiology Network (AGEN) and Diabetes Meta-analysis of Trans-ethnic Association Studies (DIAMANTE) [17]; European T2D: the data from the meta-analyses of GWAS results from 122 studies and 74,124 T2D cases and 824,006 controls of European ancestry from the DIAMANTE Consortium [18]. T2D data were downloaded from https://t2d.hugeamp.org/.

ESM Table 6 Heritability of FPG and OGTT2H in the MAGIC consortium.

Trait	MAGIC1000G_FG_EAS	MAGIC1000G_FG_EUR	MAGIC1000G_2hGlu_EAS	MAGIC1000G_2hGlu_EUR
**Trait sample size**	35619	200622	8509	63396
**Total_Observed_scale_h2_trait**	0.020(0.014)	0.047(0.008)	0.132(0.053)	0.058(0.011)
**Lambda_GC_trait**	1.014	1.127	0.993	1.053
**Mean_Chi^2_trait**	1.031	1.242	0.994	1.070
**Intercept_trait**	1.019(0.007)	1.098(0.024)	0.969(0.006)	1.019(0.008)
**Ratio_trait**	0.602(0.217)	0.405(0.100)	Ratio < 0	0.276(0.110)

ESM Table 7 Replication of the lead SNPs in the NIPT PLUS, BIGCS cohort and meta-analysis.

						Discovery study			NIPT PLUS			BIGCS			BIGCS and PLUS meta results			Discovery versus BIGCS and PLUS meta results			
Trait	SNP	CHR	BP	A1	A2	BETA	SE	*P*	BETA	SE	*P*	BETA	SE	*P*	BETA	SE	*P*	P_{diff}	Direction	*P*	P_{het}
GDM	rs10830963	11	92975544	G	C	0.449	0.04	4.42E-29	0.403	0.079	**3.82E-07**	0.376	0.097	**1.07E-04**	0.392	0.061	**1.74E-10**	**0.295**	--	**7.374E-38**	**0.434**
GDM	rs13266634	8	117172544	T	C	-0.255	0.042	1.83E-09	-0.138	0.083	9.69E-02	-0.153	0.093	9.84E-02	-0.145	0.062	**1.93E-02**	**0.135**	--	**3.328E-10**	**0.142**
GDM	rs1597916	2	206947000	C	G	0.503	0.09	1.98E-08	0.376	0.153	**1.42E-02**	0.035	0.199	8.59E-01	0.249	0.121	**4.03E-02**	**0.097**	++	**9.807E-09**	**0.092**
GDM	rs7766070	6	20686342	A	C	0.235	0.034	8.10E-12	0.13	0.071	6.86E-02	0.198	0.1	**4.83E-02**	0.153	0.058	**8.55E-03**	**0.189**	++	**5.123E-13**	**0.221**
OGTT0H	rs10476553	5	96383142	C	G	-0.139	0.011	8.97E-37	-0.103	0.026	**5.83E-05**	-0.114	0.035	**1.18E-03**	-0.107	0.021	**2.45E-07**	**0.156**	--	**2.455E-42**	**0.177**
OGTT0H	rs10823318	10	69220168	T	A	-0.087	0.013	6.68E-11	-0.09	0.031	**3.64E-03**	-0.055	0.039	1.61E-01	-0.077	0.024	**1.61E-03**	**0.373**	++	**4.385E-13**	**0.724**
OGTT0H	rs10974438	9	4291928	C	A	0.071	0.012	9.74E-10	0.053	0.028	5.61E-02	0.075	0.035	**3.34E-02**	0.062	0.022	**4.81E-03**	**0.373**	--	**1.756E-11**	**0.700**
OGTT0H	rs1912980	2	60354720	T	C	-0.077	0.012	1.15E-10	-0.084	0.028	**2.62E-03**	-0.052	0.037	1.56E-01	-0.072	0.022	**1.13E-03**	**0.392**	--	**5.13E-13**	**0.848**
OGTT0H	rs58048170	6	20665499	C	T	0.062	0.011	8.95E-09	-0.001	0.025	9.57E-01	0.055	0.035	1.16E-01	0.018	0.021	3.82E-01	**0.067**	--	**3.681E-08**	**0.060**
OGTT0H	rs59858868	9	687719	G	A	0.086	0.012	1.63E-12	0.084	0.031	**6.57E-03**	0.028	0.039	4.73E-01	0.063	0.024	**9.93E-03**	**0.273**	--	**7.648E-14**	**0.394**
OGTT0H	rs6048209	20	22590445	G	A	-0.137	0.015	4.20E-21	-0.145	0.035	**3.14E-05**	-0.128	0.046	**5.25E-03**	-0.139	0.028	**5.50E-07**	**0.398**	++	**1.258E-26**	**0.953**
OGTT0H	rs730497	7	44184122	A	G	0.173	0.017	3.27E-24	0.19	0.034	**2.95E-08**	0.074	0.044	9.46E-02	0.146	0.027	**6.14E-08**	**0.28**	++	**1.542E-30**	**0.407**
OGTT0H	rs780094	2	27518370	T	C	-0.071	0.012	1.83E-09	-0.069	0.026	**7.18E-03**	NA	NA	NA	NA	NA	NA	**NA**	--	**4.478E-11**	**0.951**
OGTT0H	rs7941837	11	92966500	T	A	0.173	0.013	5.75E-43	0.218	0.029	**7.98E-14**	0.215	0.034	**1.70E-10**	0.217	0.022	**6.86E-23**	**0.091**	--	**1.061E-63**	**0.079**
OGTT0H	rs853774	2	168956886	A	G	-0.147	0.014	2.88E-26	-0.161	0.029	**3.54E-08**	-0.084	0.034	**1.46E-02**	-0.129	0.022	**6.88E-09**	**0.312**	--	**1.425E-33**	**0.489**
OGTT1H	rs1001525	11	35413115	T	A	-0.085	0.014	3.56E-09	-0.013	0.035	7.06E-01	-0.055	0.04	1.67E-01	-0.032	0.026	2.32E-01	**0.08**	++	**8.789E-09**	**0.073**
OGTT1H	rs118083802	6	20716830	C	G	0.593	0.06	3.38E-23	0.005	0.076	9.52E-01	0.058	0.075	4.36E-01	0.032	0.054	5.51E-01	0.000	++	**1.54E-12**	0.000
OGTT1H	rs1260334	2	27525730	A	C	0.071	0.012	8.44E-10	0.037	0.026	1.57E-01	0.111	0.034	**1.20E-03**	0.064	0.021	**2.08E-03**	**0.38**	++	**6.762E-12**	**0.755**
OGTT1H	rs742762	6	39078868	C	A	-0.08	0.012	5.61E-11	-0.035	0.029	2.33E-01	-0.023	0.037	5.31E-01	-0.030	0.023	1.86E-01	**0.06**	++	**1.508E-10**	**0.053**
OGTT1H	rs7683010	4	57492982	T	G	0.059	0.011	2.79E-08	0.068	0.025	**6.79E-03**	NA	NA	NA	NA	NA	NA	**NA**	++	**6.836E-10**	**0.739**
OGTT1H	rs7941837	11	92966500	T	A	0.218	0.012	9.59E-70	0.234	0.029	**1.99E-15**	0.227	0.034	**1.79E-11**	0.231	0.022	**1.81E-25**	**0.35**	--	**6.04E-94**	**0.607**
OGTT1H	rs9650069	8	117191781	T	C	-0.115	0.018	8.65E-11	-0.085	0.031	**6.36E-03**	-0.049	0.035	1.61E-01	-0.069	0.023	**2.99E-03**	**0.118**	--	**3.268E-12**	**0.120**
OGTT2H	rs10830962	11	92965261	G	C	0.146	0.013	4.74E-30	0.146	0.029	**5.87E-07**	0.129	0.034	**1.92E-04**	0.139	0.022	**4.70E-10**	**0.384**	--	**1.363E-38**	**0.785**
OGTT2H	rs11975703	7	44109506	A	G	-0.079	0.013	3.05E-09	-0.075	0.031	**1.49E-02**	-0.091	0.04	**2.36E-02**	-0.081	0.024	**9.38E-04**	**0.398**	--	**1.115E-11**	**0.960**
OGTT2H	rs4746822	10	69223185	T	C	0.114	0.013	4.33E-18	0.152	0.031	**1.42E-06**	0.14	0.039	**3.44E-04**	0.147	0.025	**1.88E-09**	**0.196**	++	**1.001E-25**	**0.238**

OGTT2H	rs7766070	6	20686342	A	C	0.106	0.011	4.42E-23	0.071	0.027	**8.13E-03**	0.073	0.037	**4.46E-02**	0.072	0.022	**8.91E-04**	**0.151**	++	**4.098E-25**	**0.158**
FPG	rs853774	2	168956886	A	G	-0.128	0.015	1.63E-18	-0.171	0.031	**2.49E-08**	NA	NA	NA	NA	NA	NA	**0.183**	--	**4.749E-25**	**0.206**
FPG	rs1260334	2	27525730	A	C	0.069	0.012	2.05E-08	0.054	0.027	**4.46E-02**	NA	NA	NA	NA	NA	NA	**0.351**	++	**2.899E-09**	**0.609**
FPG	rs10476553	5	96383142	C	G	-0.108	0.012	7.58E-21	-0.064	0.027	**1.83E-02**	NA	NA	NA	NA	NA	NA	**0.132**	--	**1.288E-21**	**0.132**
FPG	rs147973072	6	116933819	T	C	-1.807	0.323	2.30E-08	0.199	0.149	1.82E-01	NA	NA	NA	NA	NA	NA	0.000	+-	0.2613	0.000
FPG	rs57501127	7	44221147	C	T	0.499	0.063	3.41E-15	0.198	0.084	**1.80E-02**	NA	NA	NA	NA	NA	NA	0.007	--	**1.207E-14**	0.004
FPG	rs59858868	9	687719	G	A	0.08	0.013	4.54E-10	0.087	0.033	**7.22E-03**	NA	NA	NA	NA	NA	NA	**0.391**	--	**1.13E-11**	**0.832**
FPG	rs10830962	11	92965261	G	C	0.133	0.013	6.72E-23	0.091	0.031	**3.24E-03**	NA	NA	NA	NA	NA	NA	**0.183**	--	**1.658E-24**	**0.205**
FPG	rs2305483	17	39986745	A	C	-1.301	0.236	3.82E-08	-0.043	0.194	8.23E-01	NA	NA	NA	NA	NA	NA	0.000	--	**0.0002499**	0.000
FPG	rs6036152	20	22575955	C	A	-0.119	0.015	1.09E-14	-0.114	0.036	**1.71E-03**	NA	NA	NA	NA	NA	NA	**0.396**	++	**7.053E-17**	**0.910**

P: P value of GWAS or meta analysis, values less than 0.05 are bolded. P_{diff}: P value for compared the effect estimates from our primary study (the discovery cohort) with those obtained from the BIGCS and PLUS meta-analysis P_{het}: P value for heterogeneity in SNP effects through meta-analysis, values greater than 0.05 are bolded.

ESM Table 8 The information of Lead SNPs about the five traits investigated in the study.

Trait	SNP	CHR	BP(GRCh38)	A1	A2	FRQ	INFO	BETA	SE	P	GENE	Marker	REGION	EFFECT
GDM	rs1597916	2	206947000	C	G	0.062	0.412	0.503	0.090	1.98E-08	CPO	novelty	intron_variant	MODIFIER
GDM	rs7766070	6	20686342	A	C	0.368	0.834	0.235	0.034	8.10E-12	*CDKAL1*	known	intron_variant	MODIFIER
GDM	rs13266634	8	117172544	T	C	0.436	0.538	-0.255	0.042	1.83E-09	SLC30A8	novelty	missense_variant	MODERATE
GDM	rs10830963	11	92975544	G	C	0.445	0.575	0.449	0.040	4.42E-29	MTNR1B	known	intron_variant	MODIFIER
FPG	rs853774	2	168956886	A	G	0.454	0.452	-0.128	0.015	1.63E-18	ABCB11	known	intron_variant	MODIFIER
FPG	rs1260334	2	27525730	A	C	0.488	0.651	0.069	0.012	2.05E-08	GCKR	known	downstream_gene_variant	MODIFIER
FPG	rs10476553	5	96383142	C	G	0.347	0.816	-0.108	0.012	7.58E-21	LOC101929710	known	intron_variant	MODIFIER
FPG	rs147973072	6	116933819	T	C	0.009	0.025	-1.807	0.323	2.30E-08	RFX6	known	downstream_gene_variant	MODIFIER
FPG	rs57501127	7	44221147	C	T	0.047	0.136	0.499	0.063	3.41E-15	CAMK2B	known	intron_variant	MODIFIER
FPG	rs59858868	9	687719	G	A	0.261	0.788	0.080	0.013	4.54E-10	KANK1	known	intron_variant	MODIFIER
FPG	rs10830962	11	92965261	G	C	0.451	0.534	0.133	0.013	6.72E-23	MTNR1B	known	upstream_gene_variant	MODIFIER
FPG	rs2305483	17	39986745	A	C	0.019	0.025	-1.301	0.236	3.82E-08	PSMD3	novelty	intron_variant	MODIFIER
FPG	rs6036152	20	22575955	C	A	0.154	0.782	-0.119	0.015	1.09E-14	LINC00261	known	intron_variant	MODIFIER
OGTT0H	rs853774	2	168956886	A	G	0.454	0.452	-0.147	0.014	2.88E-26	ABCB11	known	intron_variant	MODIFIER
OGTT0H	rs1912980	2	60354720	T	C	0.333	0.685	-0.077	0.012	1.15E-10	MIR4432HG	known	downstream_gene_variant	MODIFIER
OGTT0H	rs780094	2	27518370	T	C	0.525	0.636	-0.071	0.012	1.83E-09	GCKR	known	intron_variant	MODIFIER
OGTT0H	rs10476553	5	96383142	C	G	0.347	0.816	-0.139	0.011	8.97E-37	LOC101929710	known	intron_variant	MODIFIER
OGTT0H	rs58048170	6	20665499	C	T	0.380	0.834	0.062	0.011	8.95E-09	CDKAL1	known	intron_variant	MODIFIER
OGTT0H	rs730497	7	44184122	A	G	0.189	0.503	0.173	0.017	3.27E-24	GCK	known	intron_variant	MODIFIER
OGTT0H	rs59858868	9	687719	G	A	0.261	0.788	0.086	0.012	1.63E-12	KANK1	known	intron_variant	MODIFIER
OGTT0H	rs10974438	9	4291928	C	A	0.377	0.685	0.071	0.012	9.74E-10	GLIS3	known	intron_variant	MODIFIER
OGTT0H	rs10823318	10	69220168	T	A	0.269	0.615	-0.087	0.013	6.68E-11	HKDC1	novelty	upstream_gene_variant	MODIFIER
OGTT0H	rs7941837	11	92966500	T	A	0.438	0.560	0.173	0.013	5.75E-43	MTNR1B	known	upstream_gene_variant	MODIFIER
OGTT0H	rs6048209	20	22590445	G	A	0.149	0.801	-0.137	0.015	4.20E-21	FOXA2	known	upstream_gene_variant	MODIFIER
OGTT1H	rs1260334	2	27525730	A	C	0.488	0.651	0.071	0.012	8.44E-10	GCKR	novelty	downstream_gene_variant	MODIFIER
OGTT1H	rs7683010	4	57492982	T	G	0.435	0.761	0.059	0.011	2.79E-08	LOC101928851-LOC105377671	novelty	intergenic_region	MODIFIER
OGTT1H	rs118083802	6	20716830	C	G	0.065	0.100	0.593	0.060	3.38E-23	CDKAL1	novelty	intron_variant	MODIFIER

OGTT1H	rs742762	6	39078868	C	A	0.331	0.648	-0.080	0.012	5.61E-11	GLP1R	novelty	intron_variant	MODIFIER
OGTT1H	rs9650069	8	117191781	T	C	0.279	0.339	-0.115	0.018	8.65E-11	SLC30A8-MED30	novelty	intergenic_region	MODIFIER
OGTT1H	rs7941837	11	92966500	T	A	0.438	0.560	0.218	0.012	9.59E-70	MTNR1B	novelty	upstream_gene_variant	MODIFIER
OGTT1H	rs1001525	11	35413115	T	A	0.218	0.608	-0.085	0.014	3.56E-09	SLC1A2	novelty	intron_variant	MODIFIER
OGTT2H	rs7766070	6	20686342	A	C	0.368	0.834	0.106	0.011	4.42E-23	CDKAL1	novelty	intron_variant	MODIFIER
OGTT2H	rs11975703	7	44109506	A	G	0.252	0.645	-0.079	0.013	3.05E-09	MIR4649	known	upstream_gene_variant	MODIFIER
OGTT2H	rs4746822	10	69223185	T	C	0.274	0.615	0.114	0.013	4.33E-18	HKDC1	novelty	intron_variant	MODIFIER
OGTT2H	rs10830962	11	92965261	G	C	0.451	0.534	0.146	0.013	4.74E-30	MTNR1B	novelty	upstream_gene_variant	MODIFIER

Lead SNP information for Figure 3. CHR: chromosome; BP(GRCh38):position, the version of position is on human GRCh38; A1: the effect allele; FRQ: the frequency of effect allele; INFO indicates the information score in the imputation process; BETA refers to the effect size in the gwas regression model; The ‘Marker’ column indicates whether the SNP was reported in the previous study, reported as known, has not been reported as novel. The ‘REGION’ indicates the SNP function region on chromosome, ‘EFFECT’ column indicates the biological effect of SNP.

ESM Table 9 Comparison of the 13 significnat loci for FPG and OGTT2H in the MAGIC consortium East Asian population.

Trait	SNP	CHR	BP	Effect_allele	Other_allele	This study population				MAGIC_EAS_population				Meta_results			
						EAF	BETA	SE	*P*	EAF	BETA	SE	*P*	BETA	SE	*P*	Direction
FPG	rs853774	2	168956886	A	G	0.454	-0.128	0.015	1.63E-18	0.481	-0.044	0.004	2.33E-31	-0.050	0.004	6.84E-37	--
FPG	rs1260334	2	27525730	A	C	0.488	0.069	0.012	2.05E-08	0.449	0.029	0.004	2.57E-12	0.033	0.004	2.73E-17	++
FPG	rs10476553	5	96383142	C	G	0.347	-0.108	0.012	7.58E-21	0.346	-0.013	0.005	1.14E-02	-0.026	0.004	1.18E-09	--
FPG	rs147973072	6	116933819	T	C	0.009	-1.807	0.323	2.30E-08	0.021	0.020	0.027	3.78E-01	0.007	0.027	7.87E-01	-+
FPG	rs57501127	7	44221147	C	T	0.047	0.499	0.063	3.41E-15	NA	NA	NA	NA	0.499	0.063	3.23E-15	-?
FPG	rs59858868	9	687719	G	A	0.261	0.080	0.013	4.54E-10	0.210	0.021	0.005	8.58E-05	0.030	0.005	1.50E-09	--
FPG	rs10830962	11	92965261	G	C	0.451	0.133	0.013	6.72E-23	0.449	0.038	0.004	1.37E-18	0.046	0.004	6.30E-32	--
FPG	rs2305483	17	39986745	A	C	0.019	-1.301	0.236	3.82E-08	0.035	0.041	0.038	2.16E-01	0.008	0.037	8.32E-01	-+
FPG	rs6036152	20	22575955	C	A	0.154	-0.119	0.015	1.09E-14	0.154	-0.045	0.006	7.67E-14	-0.054	0.005	4.23E-24	++
OGTT2H	rs7766070	6	20686342	A	C	0.368	0.106	0.011	4.42E-23	0.406	0.118	0.032	7.48E-04	0.108	0.010	4.37E-26	++
OGTT2H	rs11975703	7	44109506	A	G	0.252	-0.079	0.013	3.05E-09	0.268	-0.028	0.045	4.07E-01	-0.075	0.013	4.53E-09	--
OGTT2H	rs4746822	10	69223185	T	C	0.274	0.114	0.013	4.33E-18	0.226	0.032	0.040	2.01E-01	0.106	0.013	2.03E-17	++
OGTT2H	rs10830962	11	92965261	G	C	0.451	0.146	0.013	4.74E-30	0.449	-0.002	0.031	9.19E-01	0.125	0.012	5.52E-26	-+

CHR: chromosome; BP(GRCh38)):position, the version of position is human GRCh38; EAF: effect allele frequency.

ESM Table 10 Detection specific SNP genetic effects of FPG lead SNPs with OGTT1H and OGTT2H

								FPG			OGTT1H				OGTT2H			
CHR	BP	SNP	A1	A2	FRQ	INFO	GENE	BETA	SE	P	BETA	SE	P	Effect_detection _$P^{FPG\ and\ OGTT1H}$	BETA	SE	P	Effect_detection _$P^{FPG\ and\ OGTT2H}$
2	168956886	rs853774	A	G	0.45	0.45	ABCB11	-0.128	0.015	1.63E-18	-0.003	0.014	8.11E-01	1.30E-09	-0.027	0.014	5.06E-02	1.31E-06
2	27525730	rs1260334	A	C	0.49	0.65	GCKR	0.069	0.012	2.05E-08	0.071	0.012	8.44E-10	3.97E-01	0.019	0.012	1.00E-01	5.38E-03
5	96383142	rs10476553	C	G	0.35	0.82	LOC101929710	-0.108	0.012	7.58E-21	-0.046	0.011	2.05E-05	1.76E-04	-0.038	0.011	4.60E-04	2.76E-05
6	116933819	rs147973072	T	C	RFX6	0.009	0.025	-1.807	0.323	2.30E-08	-1.609	0.307	1.54E-07	3.61E-01	0.095	0.307	7.57E-01	4.38E-05
7	44221147	rs57501127	C	T	CAMK2B	0.047	0.136	0.499	0.063	3.41E-15	0.194	0.059	1.04E-03	8.09E-04	0.222	0.060	2.27E-04	2.52E-03
9	687719	rs59858868	G	A	0.26	0.79	KANK1	0.080	0.013	4.54E-10	-0.023	0.012	5.10E-02	1.14E-08	-0.023	0.012	5.63E-02	1.53E-08
11	92965261	rs10830962	G	C	0.45	0.53	MTNR1B	0.133	0.013	6.72E-23	0.222	0.013	1.12E-69	3.52E-06	0.146	0.013	4.74E-30	3.16E-01
17	39986745	rs2305483	A	C	PSMD3	0.019	0.025	-1.301	0.236	3.82E-08	0.241	0.220	2.73E-01	4.41E-06	-0.110	0.224	6.21E-01	4.91E-04
20	22575955	rs6036152	C	A	0.15	0.78	LINC00261	-0.119	0.015	1.09E-14	-0.050	0.014	5.23E-04	1.81E-03	-0.064	0.015	1.22E-05	1.36E-02

Effect_detection _$P^{FPG\ and\ OGTT1H}$: two-sample *t*-test *P*-value of difference in specific SNP genetic effects between FPG and OGTT1H GWAS; Effect_detection _$P^{FPG\ and\ OGTT2H}$: two-sample *t*-test *P*-value of difference in specific SNP genetic effects between FPG and OGTT2H. A *P*-value less than 0.05 suggests a statistically significant difference in the effect of the Single Nucleotide Polymorphism (SNP) . Details of the two-sample *t*-test can be referred to Supplementary Notes.

ESM Table 11 MR results of GDM and 4 quantitative glycemic traits and biomarkers.

exposure	outcome	method	nsnp	b	se	pval	method_het	Q	Q_pval	egger_intercept	pleio_se	pleio_p
Absolute_neutrophils	GDM	MR Egger	17	-0.283	0.079	2.84E-03	MR Egger	10.864	0.762	0.015	0.031	0.629
Absolute_neutrophils	GDM	Weighted median	17	-0.276	0.081	6.75E-04	MR Egger	10.864	0.762	0.015	0.031	0.629
Absolute_neutrophils	GDM	Inverse variance weighted	17	-0.260	0.065	6.57E-05	MR Egger	10.864	0.762	0.015	0.031	0.629
Absolute_neutrophils	GDM	Simple mode	17	-0.271	0.111	2.65E-02	MR Egger	10.864	0.762	0.015	0.031	0.629
Absolute_neutrophils	GDM	Weighted mode	17	-0.292	0.086	3.71E-03	MR Egger	10.864	0.762	0.015	0.031	0.629
Lymphocyte_percentage	GDM	MR Egger	9	0.512	0.129	5.30E-03	Inverse variance weighted	3.300	0.914	-0.031	0.029	0.332
Lymphocyte_percentage	GDM	Weighted median	9	0.471	0.127	2.01E-04	Inverse variance weighted	3.300	0.914	-0.031	0.029	0.332
Lymphocyte_percentage	GDM	Inverse variance weighted	9	0.436	0.106	3.74E-05	Inverse variance weighted	3.300	0.914	-0.031	0.029	0.332
Lymphocyte_percentage	GDM	Simple mode	9	0.491	0.161	1.57E-02	Inverse variance weighted	3.300	0.914	-0.031	0.029	0.332
Lymphocyte_percentage	GDM	Weighted mode	9	0.491	0.158	1.44E-02	Inverse variance weighted	3.300	0.914	-0.031	0.029	0.332
Neutrophil_percentage	GDM	MR Egger	10	-0.481	0.121	4.14E-03	MR Egger	8.211	0.413	0.020	0.032	0.550
Neutrophil_percentage	GDM	Weighted median	10	-0.447	0.126	3.82E-04	MR Egger	8.211	0.413	0.020	0.032	0.550
Neutrophil_percentage	GDM	Inverse variance weighted	10	-0.438	0.099	9.47E-06	MR Egger	8.211	0.413	0.020	0.032	0.550
Neutrophil_percentage	GDM	Simple mode	10	-0.470	0.155	1.42E-02	MR Egger	8.211	0.413	0.020	0.032	0.550
Neutrophil_percentage	GDM	Weighted mode	10	-0.470	0.148	1.14E-02	MR Egger	8.211	0.413	0.020	0.032	0.550
White_blood_cell	GDM	MR Egger	23	-0.249	0.071	1.99E-03	MR Egger	13.238	0.900	0.009	0.019	0.645
White_blood_cell	GDM	Weighted median	23	-0.268	0.079	6.51E-04	MR Egger	13.238	0.900	0.009	0.019	0.645
White_blood_cell	GDM	Inverse variance weighted	23	-0.233	0.061	1.38E-04	MR Egger	13.238	0.900	0.009	0.019	0.645
White_blood_cell	GDM	Simple mode	23	-0.257	0.111	2.98E-02	MR Egger	13.238	0.900	0.009	0.019	0.645
White_blood_cell	GDM	Weighted mode	23	-0.281	0.080	2.04E-03	MR Egger	13.238	0.900	0.009	0.019	0.645

Method_het and Q_pval refer to the model of heterogeneity test and p value of statistic test; egger_intercept, pleio_se,pleio_p refer to the intercept,se and P value of pleiotropy test.

ESM Table 12 Instrumental variables of biomarkers as exposure and GDM as outcome.

exposure	outcome	SNP	effect_allele	other_allele	eaf	beta.exposure	se.exposure	pval.exposure	samplesize.exposure	R2	F	beta.outcome	se.outcome	pval.outcome	samplesize.outcome
Absolute_neutrophils	GDM	rs116877864	C	G	0.02	-2.75	0.15	8.29E-78	28541	0.012	350.80	1.07	0.54	0.05	22882
Absolute_neutrophils	GDM	rs117264195	C	T	0.04	-0.71	0.10	1.11E-12	28541	0.002	50.68	-0.19	0.37	0.61	22882
Absolute_neutrophils	GDM	rs118092418	A	G	0.04	1.29	0.08	2.20E-52	28541	0.008	232.93	-0.11	0.31	0.73	22882
Absolute_neutrophils	GDM	rs141690080	G	A	0.02	2.07	0.23	6.47E-19	28541	0.003	79.03	0.66	0.85	0.44	22882
Absolute_neutrophils	GDM	rs142327763	G	C	0.04	1.58	0.09	2.04E-74	28541	0.012	335.05	-0.43	0.32	0.18	22882
Absolute_neutrophils	GDM	rs142772971	G	T	0.02	0.78	0.11	1.94E-13	28541	0.002	54.12	-0.12	0.39	0.77	22882
Absolute_neutrophils	GDM	rs143437186	T	C	0.01	1.85	0.33	2.83E-08	28541	0.001	30.84	-1.84	1.23	0.13	22882
Absolute_neutrophils	GDM	rs144745936	A	T	0.02	3.17	0.16	1.08E-89	28541	0.014	406.12	-1.03	0.59	0.08	22882
Absolute_neutrophils	GDM	rs181359740	T	C	0.03	2.88	0.12	1.68E-117	28541	0.018	536.02	-0.76	0.46	0.10	22882
Absolute_neutrophils	GDM	rs184338334	G	A	0.02	0.81	0.14	2.28E-08	28541	0.001	31.25	-0.96	0.53	0.07	22882
Absolute_neutrophils	GDM	rs191558349	A	G	0.04	-0.70	0.11	1.98E-10	28541	0.001	40.51	-0.37	0.40	0.36	22882
Absolute_neutrophils	GDM	rs2305483	A	C	0.02	3.73	0.20	5.46E-80	28541	0.012	360.94	-1.20	0.73	0.10	22882
Absolute_neutrophils	GDM	rs7021	A	T	0.46	0.21	0.01	6.29E-120	28541	0.019	547.39	-0.06	0.03	0.08	22882
Absolute_neutrophils	GDM	rs76714842	A	G	0.19	0.09	0.01	3.72E-14	28541	0.002	57.37	0.00	0.04	0.93	22882
Absolute_neutrophils	GDM	rs77115032	G	A	0.02	0.72	0.10	3.59E-12	28541	0.002	48.38	-0.22	0.38	0.55	22882
Absolute_neutrophils	GDM	rs77474927	T	C	0.03	0.62	0.12	8.69E-08	28541	0.001	28.66	-0.55	0.43	0.19	22882
Absolute_neutrophils	GDM	rs78431470	T	G	0.09	-0.19	0.04	1.02E-07	28541	0.001	28.35	-0.06	0.14	0.64	22882
Lymphocyte_percentage	GDM	rs116877864	C	G	0.02	1.78	0.15	4.66E-33	28341	0.005	143.82	1.07	0.54	0.05	22882
Lymphocyte_percentage	GDM	rs118092418	A	G	0.04	-0.82	0.08	6.32E-22	28341	0.003	92.77	-0.11	0.31	0.73	22882
Lymphocyte_percentage	GDM	rs142327763	G	C	0.04	-0.94	0.09	4.49E-27	28341	0.004	116.35	-0.43	0.32	0.18	22882
Lymphocyte_percentage	GDM	rs150194598	A	G	0.03	-2.66	0.18	5.41E-48	28341	0.007	212.65	-1.47	0.67	0.03	22882
Lymphocyte_percentage	GDM	rs16851441	G	A	0.24	0.07	0.01	1.47E-08	28341	0.001	32.11	-0.03	0.04	0.48	22882
Lymphocyte_percentage	GDM	rs181359740	T	C	0.03	-1.79	0.13	7.58E-46	28341	0.007	202.73	-0.76	0.46	0.10	22882
Lymphocyte_percentage	GDM	rs2305483	A	C	0.02	-2.42	0.20	5.19E-34	28341	0.005	148.20	-1.20	0.73	0.10	22882
Lymphocyte_percentage	GDM	rs4795420	G	A	0.46	-0.14	0.01	3.56E-48	28341	0.007	213.49	-0.06	0.03	0.06	22882

Lymphocyte_percentage	GDM	rs7752721	T	C	0.07	0.09	0.02	2.72E-08	28341	0.001	30.91	0.02	0.06	0.79	22882
Neutrophil_percentage	GDM	rs116877864	C	G	0.02	-1.86	0.15	1.10E-35	28456	0.005	155.90	1.07	0.54	0.05	22882
Neutrophil_percentage	GDM	rs118092418	A	G	0.04	0.86	0.08	2.13E-24	28456	0.004	104.08	-0.11	0.31	0.73	22882
Neutrophil_percentage	GDM	rs141690080	G	A	0.02	1.34	0.23	1.02E-08	28456	0.001	32.83	0.66	0.85	0.44	22882
Neutrophil_percentage	GDM	rs142327763	G	C	0.04	1.00	0.09	1.12E-30	28456	0.005	132.88	-0.43	0.32	0.18	22882
Neutrophil_percentage	GDM	rs142470396	G	A	0.04	-0.39	0.06	9.33E-10	28456	0.001	37.48	0.62	0.22	0.01	22882
Neutrophil_percentage	GDM	rs150194598	A	G	0.03	2.82	0.18	5.38E-54	28456	0.008	240.38	-1.47	0.67	0.03	22882
Neutrophil_percentage	GDM	rs181359740	T	C	0.03	1.91	0.13	4.20E-52	28456	0.008	231.63	-0.76	0.46	0.10	22882
Neutrophil_percentage	GDM	rs2305483	A	C	0.02	2.49	0.20	4.11E-36	28456	0.006	157.87	-1.20	0.73	0.10	22882
Neutrophil_percentage	GDM	rs4795420	G	A	0.46	0.14	0.01	3.51E-54	28456	0.008	241.24	-0.06	0.03	0.06	22882
Neutrophil_percentage	GDM	rs80347875	T	A	0.20	-0.07	0.01	1.26E-08	28456	0.001	32.42	-0.02	0.04	0.65	22882
White_blood_cell	GDM	rs116877864	C	G	0.02	-2.87	0.15	3.58E-85	28477	0.013	385.07	1.07	0.54	0.05	22882
White_blood_cell	GDM	rs117264195	C	T	0.04	-0.78	0.10	3.72E-15	28477	0.002	61.91	-0.19	0.37	0.61	22882
White_blood_cell	GDM	rs118092418	A	G	0.04	1.32	0.08	1.43E-55	28477	0.009	247.67	-0.11	0.31	0.73	22882
White_blood_cell	GDM	rs12672925	T	C	0.06	-0.22	0.04	1.27E-07	28477	0.001	27.93	-0.04	0.15	0.81	22882
White_blood_cell	GDM	rs141690080	G	A	0.02	2.02	0.23	2.67E-18	28477	0.003	76.22	0.66	0.85	0.44	22882
White_blood_cell	GDM	rs142327763	G	C	0.04	1.61	0.09	8.17E-78	28477	0.012	350.84	-0.43	0.32	0.18	22882
White_blood_cell	GDM	rs142772971	G	T	0.02	0.82	0.11	8.27E-15	28477	0.002	60.33	-0.12	0.39	0.77	22882
White_blood_cell	GDM	rs143437186	T	C	0.01	1.91	0.33	9.55E-09	28477	0.001	32.95	-1.84	1.23	0.13	22882
White_blood_cell	GDM	rs144745936	A	T	0.02	3.27	0.16	7.09E-96	28477	0.015	434.95	-1.03	0.59	0.08	22882
White_blood_cell	GDM	rs181359740	T	C	0.03	2.94	0.12	4.28E-124	28477	0.020	566.94	-0.76	0.46	0.10	22882
White_blood_cell	GDM	rs184338334	G	A	0.02	0.83	0.14	8.25E-09	28477	0.001	33.23	-0.96	0.53	0.07	22882
White_blood_cell	GDM	rs191558349	A	G	0.04	-0.77	0.11	1.40E-12	28477	0.002	50.23	-0.37	0.40	0.36	22882
White_blood_cell	GDM	rs2299002	C	A	0.27	-0.06	0.01	1.67E-09	28477	0.001	36.34	0.00	0.04	0.98	22882
White_blood_cell	GDM	rs2305483	A	C	0.02	3.95	0.20	1.69E-90	28477	0.014	409.88	-1.20	0.73	0.10	22882
White_blood_cell	GDM	rs3760531	C	G	0.02	-0.89	0.15	1.68E-09	28477	0.001	36.34	-0.03	0.55	0.96	22882
White_blood_cell	GDM	rs6989110	C	T	0.30	-0.07	0.01	1.11E-08	28477	0.001	32.66	0.02	0.04	0.59	22882
White_blood_cell	GDM	rs7021	A	T	0.46	0.22	0.01	1.79E-129	28477	0.020	592.17	-0.06	0.03	0.08	22882
White_blood_cell	GDM	rs76677887	C	T	0.20	0.09	0.01	9.69E-16	28477	0.002	64.56	0.00	0.04	0.99	22882

White_blood_cell	GDM	rs7692	A	C	0.31	-0.06	0.01	1.50E-08	28477	0.001	32.07	0.01	0.04	0.76	22882
White_blood_cell	GDM	rs77115032	G	A	0.02	0.74	0.10	5.20E-13	28477	0.002	52.17	-0.22	0.38	0.55	22882
White_blood_cell	GDM	rs77474927	T	C	0.03	0.64	0.12	3.76E-08	28477	0.001	30.28	-0.55	0.43	0.19	22882
White_blood_cell	GDM	rs78431470	T	G	0.09	-0.21	0.04	7.81E-09	28477	0.001	33.34	-0.06	0.14	0.64	22882
White_blood_cell	GDM	rs79540179	C	T	0.01	-3.30	0.43	3.24E-14	28477	0.002	57.64	-1.13	1.60	0.48	22882

$$F = \frac{N - k - 1}{k} \times \frac{R^2}{1 - R^2}$$

$$R^2 = \frac{2 * \beta^2 * MAF * (1 - MAF)}{2 * \beta^2 * MAF * (1 - MAF) + se^2 * 2 * N * MAF * (1 - MAF)}$$

ESM Table 13 The power of Mendelian Randomization(MR) analyses.

Exposure	Outcome	Sample size	α	K	OR	R^2	Power	F-statistics
Absolute_neutrophils	GDM	22882	0.05	0.14	0.77	0.116	0.99	3003.62
Lymphocyte_percentage	GDM	22882	0.05	0.14	1.55	0.042	1	1004.18
Neutrophil_percentage	GDM	22882	0.05	0.14	0.65	0.048	0.99	1154.71
White_blood_cell	GDM	22882	0.05	0.14	0.79	0.128	0.98	3359.83

α: type I error rate
K: proportion of cases in the study
OR: true odds ratio of the outcome variable per standard deviation of the exposure variable
R^2: Proportion of variance explained for the association between the SNP or allele score (Z) and the exposure variable (X)
F-statistic: the strength of the instrument

ESM Table 14 MR results of GDM and 4 quantitative glycemic traits and 4 biomarkers use meta data with PLUS cohort.

exposure	outcome	method	nsnp	b	se	pval	method_het	Q	Q_df	Q_pval	egger_intercept	pleio_se	pleio_p
Absolute_neutrophils	GDM	MR Egger	16	-0.243	0.082	1.05E-02	MR Egger	11.00705288	14	0.685	0.002	0.014	0.866
Absolute_neutrophils	GDM	Weighted median	16	-0.219	0.086	1.10E-02	MR Egger	11.00705288	14	0.685	0.002	0.014	0.866
Absolute_neutrophils	GDM	Inverse variance weighted	16	-0.235	0.067	4.44E-04	MR Egger	11.00705288	14	0.685	0.002	0.014	0.866
Absolute_neutrophils	GDM	Simple mode	16	-0.191	0.121	1.36E-01	MR Egger	11.00705288	14	0.685	0.002	0.014	0.866
Absolute_neutrophils	GDM	Weighted mode	16	-0.215	0.087	2.65E-02	MR Egger	11.00705288	14	0.685	0.002	0.014	0.866
Lymphocyte_percentage	GDM	MR Egger	12	0.558	0.154	4.71E-03	Inverse variance weighted	17.43531351	11	0.096	-0.059	0.021	0.019
Lymphocyte_percentage	GDM	Weighted median	12	0.352	0.144	1.48E-02	Inverse variance weighted	17.43531351	11	0.096	-0.059	0.021	0.019
Lymphocyte_percentage	GDM	Inverse variance weighted	12	0.247	0.135	6.69E-02	Inverse variance weighted	17.43531351	11	0.096	-0.059	0.021	0.019
Lymphocyte_percentage	GDM	Simple mode	12	0.349	0.188	9.01E-02	Inverse variance weighted	17.43531351	11	0.096	-0.059	0.021	0.019
Lymphocyte_percentage	GDM	Weighted mode	12	0.362	0.161	4.58E-02	Inverse variance weighted	17.43531351	11	0.096	-0.059	0.021	0.019
Neutrophil_percentage	GDM	MR Egger	13	-0.555	0.147	3.05E-03	Inverse variance weighted	14.62092434	12	0.263	0.047	0.021	0.044
Neutrophil_percentage	GDM	Weighted median	13	-0.349	0.136	1.06E-02	Inverse variance weighted	14.62092434	12	0.263	0.047	0.021	0.044
Neutrophil_percentage	GDM	Inverse variance weighted	13	-0.315	0.112	5.06E-03	Inverse variance weighted	14.62092434	12	0.263	0.047	0.021	0.044
Neutrophil_percentage	GDM	Simple mode	13	-0.397	0.202	7.31E-02	Inverse variance weighted	14.62092434	12	0.263	0.047	0.021	0.044
Neutrophil_percentage	GDM	Weighted mode	13	-0.377	0.147	2.45E-02	Inverse variance weighted	14.62092434	12	0.263	0.047	0.021	0.044
White_blood_cell	GDM	MR Egger	19	-0.211	0.079	1.57E-02	MR Egger	13.52139763	17	0.701	0.000	0.014	0.979
White_blood_cell	GDM	Weighted median	19	-0.211	0.085	1.36E-02	MR Egger	13.52139763	17	0.701	0.000	0.014	0.979
White_blood_cell	GDM	Inverse variance weighted	19	-0.210	0.063	8.97E-04	MR Egger	13.52139763	17	0.701	0.000	0.014	0.979
White_blood_cell	GDM	Simple mode	19	-0.161	0.131	2.34E-01	MR Egger	13.52139763	17	0.701	0.000	0.014	0.979
White_blood_cell	GDM	Weighted mode	19	-0.213	0.087	2.46E-02	MR Egger	13.52139763	17	0.701	0.000	0.014	0.979
Absolute_neutrophils	FPG	MR Egger	16	-0.256	0.029	3.90E-07	MR Egger	14.10923329	14	0.442	0.004	0.005	0.392
Absolute_neutrophils	FPG	Weighted median	16	-0.236	0.034	3.12E-12	MR Egger	14.10923329	14	0.442	0.004	0.005	0.392
Absolute_neutrophils	FPG	Inverse variance weighted	16	-0.241	0.023	1.91E-25	MR Egger	14.10923329	14	0.442	0.004	0.005	0.392
Absolute_neutrophils	FPG	Simple mode	16	-0.265	0.050	8.88E-05	MR Egger	14.10923329	14	0.442	0.004	0.005	0.392
Absolute_neutrophils	FPG	Weighted mode	16	-0.255	0.038	7.04E-06	MR Egger	14.10923329	14	0.442	0.004	0.005	0.392
Lymphocyte_percentage	FPG	MR Egger	12	0.457	0.067	4.59E-05	Inverse variance weighted	27.04584468	11	0.005	-0.025	0.009	0.021

Lymphocyte_percentage	FPG	Weighted median	12	0.372	0.055	1.41E-11	Inverse variance weighted	27.04584468	11	0.005	-0.025	0.009	0.021
Lymphocyte_percentage	FPG	Inverse variance weighted	12	0.324	0.058	2.22E-08	Inverse variance weighted	27.04584468	11	0.005	-0.025	0.009	0.021
Lymphocyte_percentage	FPG	Simple mode	12	0.379	0.088	1.19E-03	Inverse variance weighted	27.04584468	11	0.005	-0.025	0.009	0.021
Lymphocyte_percentage	FPG	Weighted mode	12	0.389	0.062	6.45E-05	Inverse variance weighted	27.04584468	11	0.005	-0.025	0.009	0.021
Neutrophil_percentage	FPG	MR Egger	13	-0.422	0.056	1.18E-05	Inverse variance weighted	18.83611261	12	0.093	0.017	0.008	0.054
Neutrophil_percentage	FPG	Weighted median	13	-0.359	0.050	5.20E-13	Inverse variance weighted	18.83611261	12	0.093	0.017	0.008	0.054
Neutrophil_percentage	FPG	Inverse variance weighted	13	-0.333	0.044	3.30E-14	Inverse variance weighted	18.83611261	12	0.093	0.017	0.008	0.054
Neutrophil_percentage	FPG	Simple mode	13	-0.393	0.088	7.31E-04	Inverse variance weighted	18.83611261	12	0.093	0.017	0.008	0.054
Neutrophil_percentage	FPG	Weighted mode	13	-0.389	0.062	4.33E-05	Inverse variance weighted	18.83611261	12	0.093	0.017	0.008	0.054
White_blood_cell	FPG	MR Egger	19	-0.227	0.028	2.48E-07	MR Egger	17.10533285	17	0.447	0.003	0.005	0.581
White_blood_cell	FPG	Weighted median	19	-0.221	0.032	3.05E-12	MR Egger	17.10533285	17	0.447	0.003	0.005	0.581
White_blood_cell	FPG	Inverse variance weighted	19	-0.217	0.022	2.76E-23	MR Egger	17.10533285	17	0.447	0.003	0.005	0.581
White_blood_cell	FPG	Simple mode	19	-0.252	0.051	1.04E-04	MR Egger	17.10533285	17	0.447	0.003	0.005	0.581
White_blood_cell	FPG	Weighted mode	19	-0.231	0.034	2.49E-06	MR Egger	17.10533285	17	0.447	0.003	0.005	0.581
Absolute_neutrophils	OGTT0H	MR Egger	16	-0.197	0.031	1.62E-05	Inverse variance weighted	21.12330653	15	0.133	-0.008	0.005	0.128
Absolute_neutrophils	OGTT0H	Weighted median	16	-0.190	0.030	1.70E-10	Inverse variance weighted	21.12330653	15	0.133	-0.008	0.005	0.128
Absolute_neutrophils	OGTT0H	Inverse variance weighted	16	-0.226	0.026	3.28E-18	Inverse variance weighted	21.12330653	15	0.133	-0.008	0.005	0.128
Absolute_neutrophils	OGTT0H	Simple mode	16	-0.249	0.044	5.08E-05	Inverse variance weighted	21.12330653	15	0.133	-0.008	0.005	0.128
Absolute_neutrophils	OGTT0H	Weighted mode	16	-0.197	0.030	9.50E-06	Inverse variance weighted	21.12330653	15	0.133	-0.008	0.005	0.128
Lymphocyte_percentage	OGTT0H	MR Egger	12	0.413	0.065	8.40E-05	Inverse variance weighted	33.35787269	11	0.000	-0.029	0.009	0.008
Lymphocyte_percentage	OGTT0H	Weighted median	12	0.298	0.048	4.45E-10	Inverse variance weighted	33.35787269	11	0.000	-0.029	0.009	0.008
Lymphocyte_percentage	OGTT0H	Inverse variance weighted	12	0.258	0.061	2.48E-05	Inverse variance weighted	33.35787269	11	0.000	-0.029	0.009	0.008
Lymphocyte_percentage	OGTT0H	Simple mode	12	0.313	0.067	7.00E-04	Inverse variance weighted	33.35787269	11	0.000	-0.029	0.009	0.008
Lymphocyte_percentage	OGTT0H	Weighted mode	12	0.306	0.052	1.02E-04	Inverse variance weighted	33.35787269	11	0.000	-0.029	0.009	0.008
Neutrophil_percentage	OGTT0H	MR Egger	13	-0.374	0.049	9.60E-06	Inverse variance weighted	15.0566031	12	0.238	0.017	0.007	0.029
Neutrophil_percentage	OGTT0H	Weighted median	13	-0.305	0.047	1.31E-10	Inverse variance weighted	15.0566031	12	0.238	0.017	0.007	0.029
Neutrophil_percentage	OGTT0H	Inverse variance weighted	13	-0.285	0.037	2.19E-14	Inverse variance weighted	15.0566031	12	0.238	0.017	0.007	0.029
Neutrophil_percentage	OGTT0H	Simple mode	13	-0.325	0.076	1.11E-03	Inverse variance weighted	15.0566031	12	0.238	0.017	0.007	0.029
Neutrophil_percentage	OGTT0H	Weighted mode	13	-0.299	0.051	7.39E-05	Inverse variance weighted	15.0566031	12	0.238	0.017	0.007	0.029

White_blood_cell	OGTT0H	MR Egger	19	-0.185	0.030	1.05E-05	Inverse variance weighted	25.24771879	18	0.118	-0.007	0.005	0.167
White_blood_cell	OGTT0H	Weighted median	19	-0.183	0.029	1.23E-10	Inverse variance weighted	25.24771879	18	0.118	-0.007	0.005	0.167
White_blood_cell	OGTT0H	Inverse variance weighted	19	-0.212	0.025	9.32E-18	Inverse variance weighted	25.24771879	18	0.118	-0.007	0.005	0.167
White_blood_cell	OGTT0H	Simple mode	19	-0.235	0.042	2.64E-05	Inverse variance weighted	25.24771879	18	0.118	-0.007	0.005	0.167
White_blood_cell	OGTT0H	Weighted mode	19	-0.184	0.029	6.33E-06	Inverse variance weighted	25.24771879	18	0.118	-0.007	0.005	0.167
Absolute_neutrophils	OGTT1H	MR Egger	16	0.057	0.027	5.26E-02	Inverse variance weighted	15.63126803	15	0.407	-0.009	0.005	0.079
Absolute_neutrophils	OGTT1H	Weighted median	16	0.041	0.028	1.46E-01	Inverse variance weighted	15.63126803	15	0.407	-0.009	0.005	0.079
Absolute_neutrophils	OGTT1H	Inverse variance weighted	16	0.027	0.022	2.28E-01	Inverse variance weighted	15.63126803	15	0.407	-0.009	0.005	0.079
Absolute_neutrophils	OGTT1H	Simple mode	16	0.030	0.037	4.32E-01	Inverse variance weighted	15.63126803	15	0.407	-0.009	0.005	0.079
Absolute_neutrophils	OGTT1H	Weighted mode	16	0.044	0.030	1.66E-01	Inverse variance weighted	15.63126803	15	0.407	-0.009	0.005	0.079
Lymphocyte_percentage	OGTT1H	MR Egger	12	-0.039	0.066	5.66E-01	MR Egger	16.96886353	10	0.075	-0.005	0.009	0.573
Lymphocyte_percentage	OGTT1H	Weighted median	12	-0.065	0.048	1.77E-01	MR Egger	16.96886353	10	0.075	-0.005	0.009	0.573
Lymphocyte_percentage	OGTT1H	Inverse variance weighted	12	-0.068	0.044	1.24E-01	MR Egger	16.96886353	10	0.075	-0.005	0.009	0.573
Lymphocyte_percentage	OGTT1H	Simple mode	12	-0.035	0.069	6.20E-01	MR Egger	16.96886353	10	0.075	-0.005	0.009	0.573
Lymphocyte_percentage	OGTT1H	Weighted mode	12	-0.061	0.048	2.31E-01	MR Egger	16.96886353	10	0.075	-0.005	0.009	0.573
Neutrophil_percentage	OGTT1H	MR Egger	13	0.052	0.055	3.65E-01	MR Egger	14.22135177	11	0.221	-0.003	0.008	0.722
Neutrophil_percentage	OGTT1H	Weighted median	13	0.062	0.041	1.31E-01	MR Egger	14.22135177	11	0.221	-0.003	0.008	0.722
Neutrophil_percentage	OGTT1H	Inverse variance weighted	13	0.037	0.036	3.02E-01	MR Egger	14.22135177	11	0.221	-0.003	0.008	0.722
Neutrophil_percentage	OGTT1H	Simple mode	13	0.003	0.068	9.63E-01	MR Egger	14.22135177	11	0.221	-0.003	0.008	0.722
Neutrophil_percentage	OGTT1H	Weighted mode	13	0.063	0.052	2.47E-01	MR Egger	14.22135177	11	0.221	-0.003	0.008	0.722
White_blood_cell	OGTT1H	MR Egger	19	0.064	0.026	2.54E-02	Inverse variance weighted	20.17334312	18	0.323	-0.009	0.004	0.046
White_blood_cell	OGTT1H	Weighted median	19	0.039	0.028	1.65E-01	Inverse variance weighted	20.17334312	18	0.323	-0.009	0.004	0.046
White_blood_cell	OGTT1H	Inverse variance weighted	19	0.030	0.022	1.74E-01	Inverse variance weighted	20.17334312	18	0.323	-0.009	0.004	0.046
White_blood_cell	OGTT1H	Simple mode	19	0.034	0.038	3.77E-01	Inverse variance weighted	20.17334312	18	0.323	-0.009	0.004	0.046
White_blood_cell	OGTT1H	Weighted mode	19	0.045	0.026	1.07E-01	Inverse variance weighted	20.17334312	18	0.323	-0.009	0.004	0.046
Absolute_neutrophils	OGTT2H	MR Egger	16	-0.047	0.027	1.07E-01	MR Egger	12.9342055	14	0.532	-0.004	0.005	0.457
Absolute_neutrophils	OGTT2H	Weighted median	16	-0.031	0.030	3.04E-01	MR Egger	12.9342055	14	0.532	-0.004	0.005	0.457
Absolute_neutrophils	OGTT2H	Inverse variance weighted	16	-0.060	0.022	6.95E-03	MR Egger	12.9342055	14	0.532	-0.004	0.005	0.457
Absolute_neutrophils	OGTT2H	Simple mode	16	-0.062	0.041	1.49E-01	MR Egger	12.9342055	14	0.532	-0.004	0.005	0.457

Absolute_neutrophils	OGTT2H	Weighted mode	16	-0.033	0.029	2.72E-01	MR Egger	12.9342055	14	0.532	-0.004	0.005	0.457
Lymphocyte_percentage	OGTT2H	MR Egger	12	0.098	0.057	1.19E-01	Inverse variance weighted	15.824248	11	0.148	-0.013	0.008	0.129
Lymphocyte_percentage	OGTT2H	Weighted median	12	0.046	0.046	3.16E-01	Inverse variance weighted	15.824248	11	0.148	-0.013	0.008	0.129
Lymphocyte_percentage	OGTT2H	Inverse variance weighted	12	0.029	0.042	4.98E-01	Inverse variance weighted	15.824248	11	0.148	-0.013	0.008	0.129
Lymphocyte_percentage	OGTT2H	Simple mode	12	0.051	0.064	4.45E-01	Inverse variance weighted	15.824248	11	0.148	-0.013	0.008	0.129
Lymphocyte_percentage	OGTT2H	Weighted mode	12	0.049	0.052	3.72E-01	Inverse variance weighted	15.824248	11	0.148	-0.013	0.008	0.129
Neutrophil_percentage	OGTT2H	MR Egger	13	-0.108	0.049	5.03E-02	Inverse variance weighted	12.41876009	12	0.413	0.010	0.007	0.159
Neutrophil_percentage	OGTT2H	Weighted median	13	-0.044	0.045	3.29E-01	Inverse variance weighted	12.41876009	12	0.413	0.010	0.007	0.159
Neutrophil_percentage	OGTT2H	Inverse variance weighted	13	-0.054	0.034	1.16E-01	Inverse variance weighted	12.41876009	12	0.413	0.010	0.007	0.159
Neutrophil_percentage	OGTT2H	Simple mode	13	-0.045	0.063	4.89E-01	Inverse variance weighted	12.41876009	12	0.413	0.010	0.007	0.159
Neutrophil_percentage	OGTT2H	Weighted mode	13	-0.041	0.046	3.83E-01	Inverse variance weighted	12.41876009	12	0.413	0.010	0.007	0.159
White_blood_cell	OGTT2H	MR Egger	19	-0.045	0.026	1.02E-01	MR Egger	15.89452642	17	0.531	-0.002	0.004	0.692
White_blood_cell	OGTT2H	Weighted median	19	-0.033	0.029	2.47E-01	MR Egger	15.89452642	17	0.531	-0.002	0.004	0.692
White_blood_cell	OGTT2H	Inverse variance weighted	19	-0.052	0.021	1.30E-02	MR Egger	15.89452642	17	0.531	-0.002	0.004	0.692
White_blood_cell	OGTT2H	Simple mode	19	-0.076	0.041	7.92E-02	MR Egger	15.89452642	17	0.531	-0.002	0.004	0.692
White_blood_cell	OGTT2H	Weighted mode	19	-0.042	0.027	1.36E-01	MR Egger	15.89452642	17	0.531	-0.002	0.004	0.692

Method_het and Q_pval refer to the model of heterogeneity test and p value of statistic test; egger_intercept, pleio_se,pleio_p refer to the intercept,se and P value of pleiotropy test.

Supplementary Figures

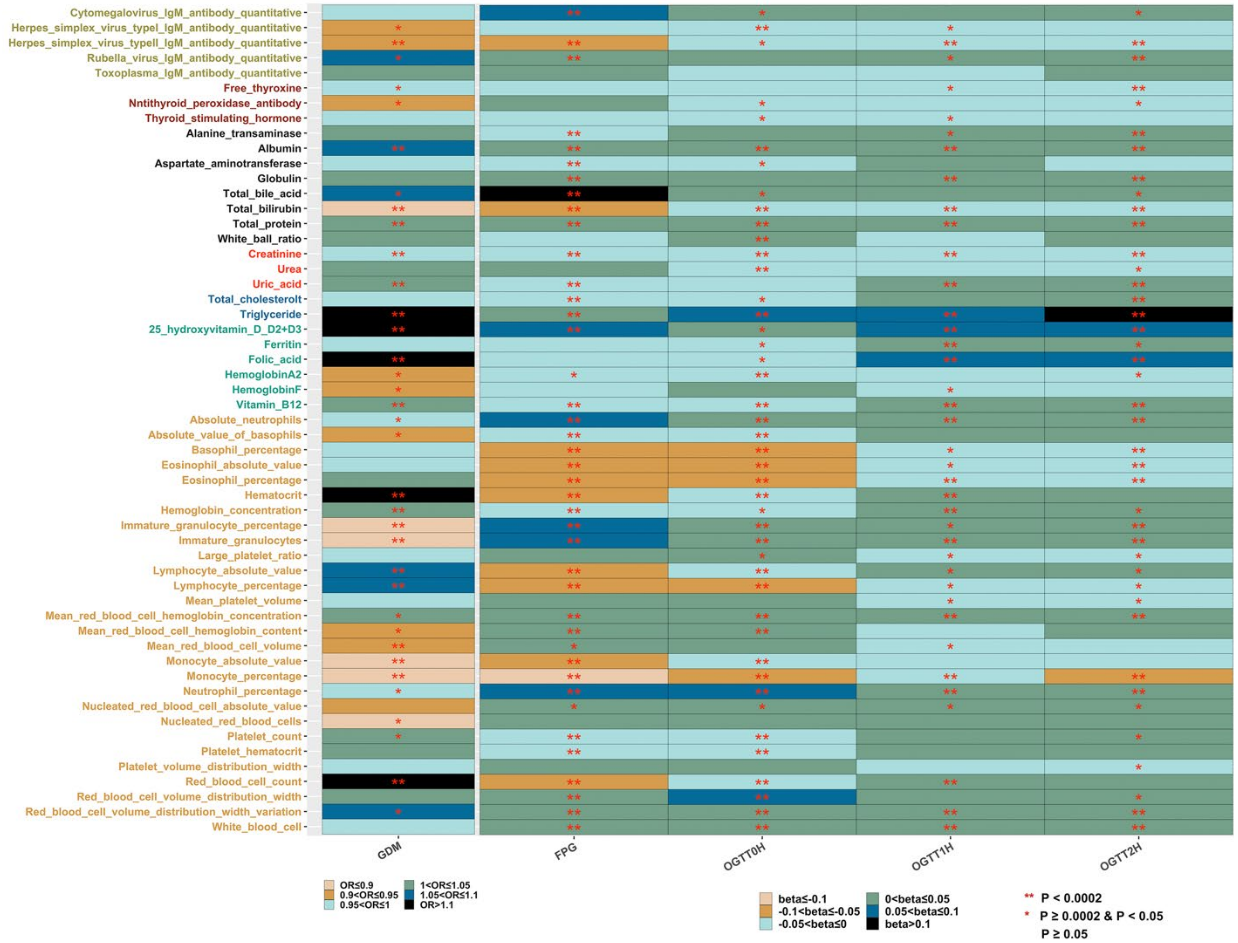

ESM Fig. 1 Multiple regression analyses of GDM and the four glycemic traits.
Standardized regression coefficients are displayed. The color of the vertical coordinate represents the categories of the biomarkers according to ESM Table 2, grass green: Infection; red-brown: Thyroid function; black: Liver function; red: Kidney function; blue: Blood lipid; cyan: Blood anemia; yellow: Blood routine. Color in square for qualitative variables indicates OR, for quantitative variables indicates effect size. The number of * sign in the square represents statistical test significance, bonferroni-adjusted threashold α is 0.0001(0.05/55/5).

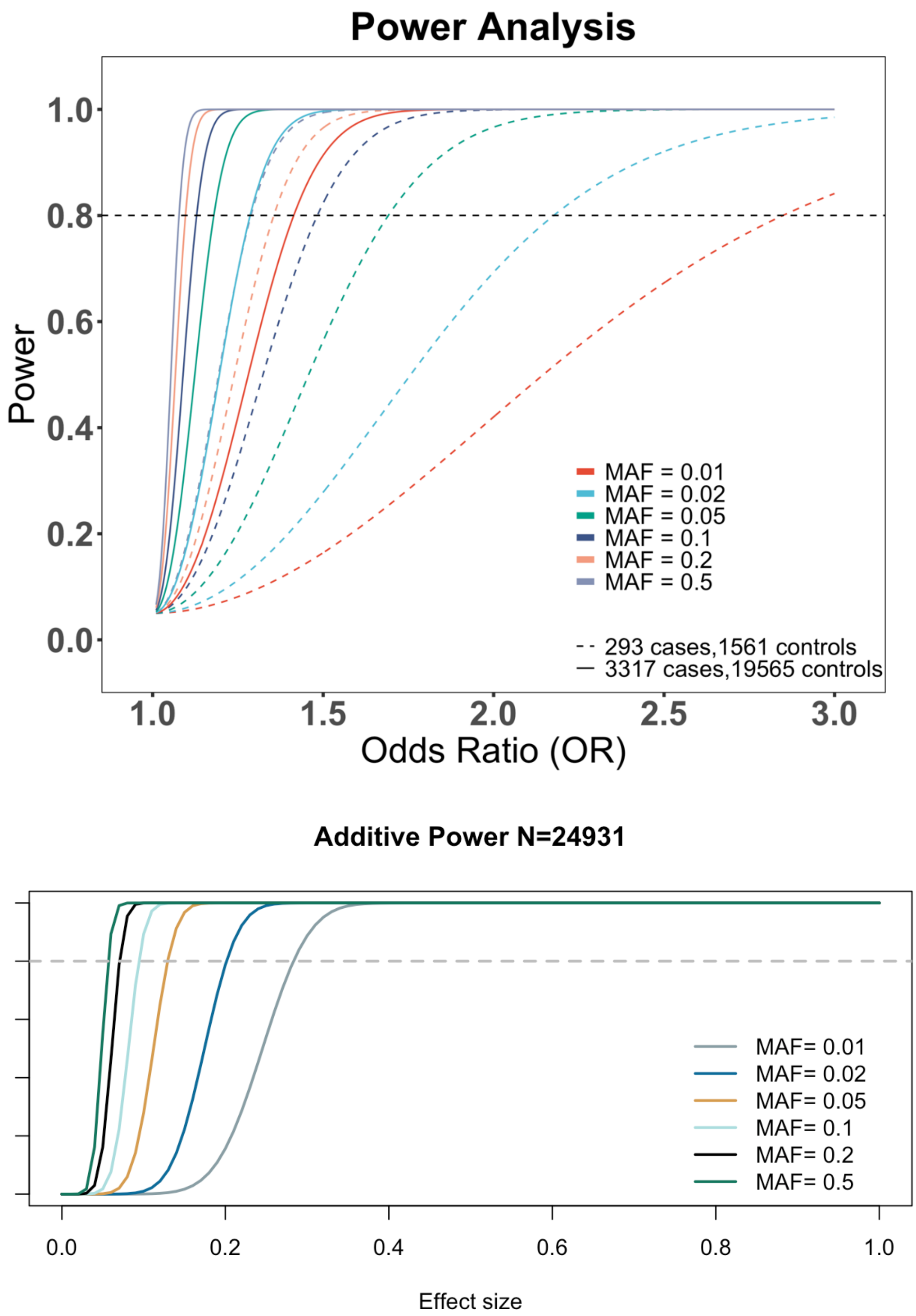

ESM Fig. 2 Power analysis of the genome-wide association analysis.

Top: Power analysis for GWAS of GDM susceptibility (3,317 cases versus 19,565 controls) mean this study and GWAS of GDM susceptibility in BIGCS replication cohort (293 cases versus 1,561 controls). Significance threshold P value < 5×10^{-8}. Bottom: Power analysis for GWAS of the four glycemic traits. Significance threshold P value < 5×10^{-8}. MAF: minor allele frequency.

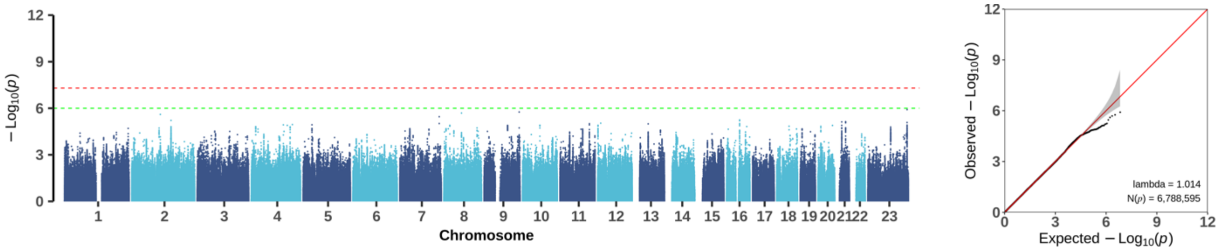

ESM Fig. 3 Manhattan plot of GDM case with medication or not (case/control=419/2898).

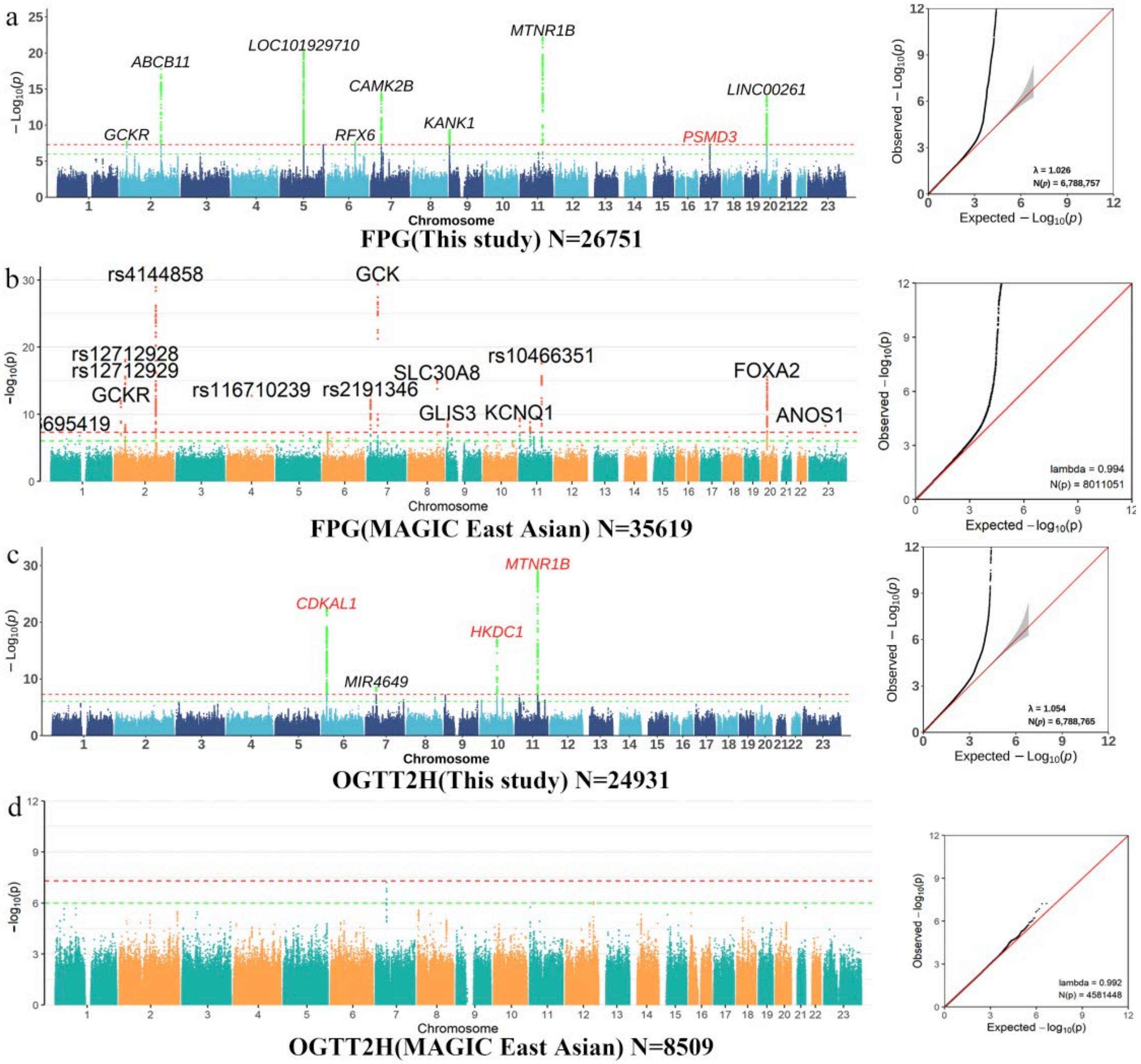

ESM Fig. 4 Comparison of the genome-wide association analysis for FPG and OGTT2H between MAGIC East Asian and our study.

For panel a and c, results from our study, newly identified loci are denoted by red signals, while loci with established knowledge are represented in black. For panel B and D, the loci were know and came from MAGIC East Asian OGTT2H and FPG GWAS summary, which were downloaded from https://magicinvestigators.org/downloads/.

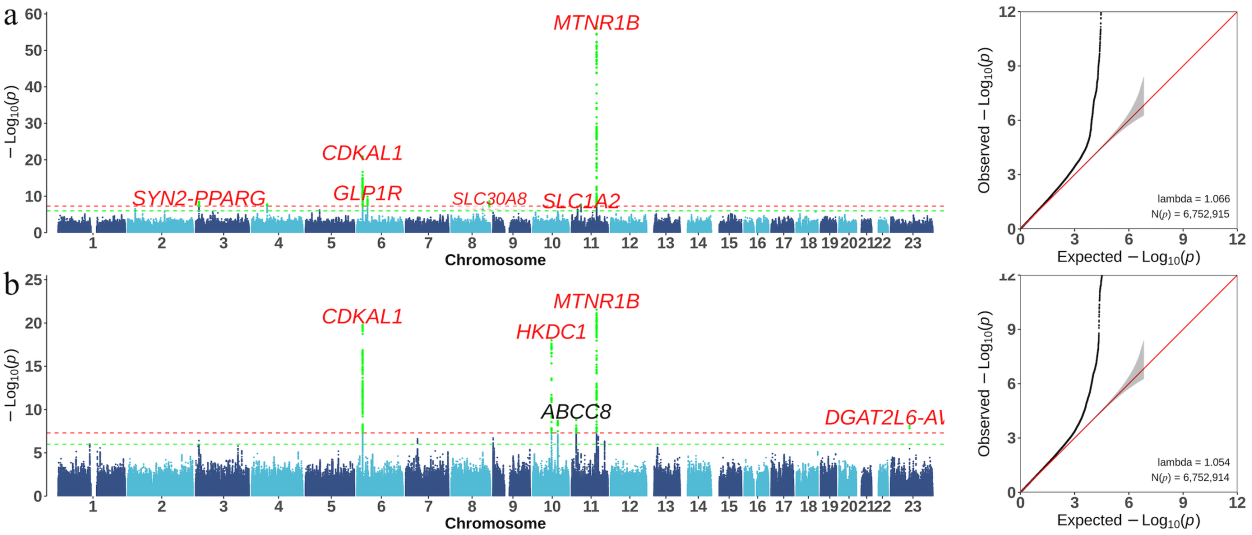

ESM Fig. 5 Manhattan and qq plot of OGTT1H(a) and OGTT2H(b) with age,BMI, gestational week of OGTT and FPG as covariants.

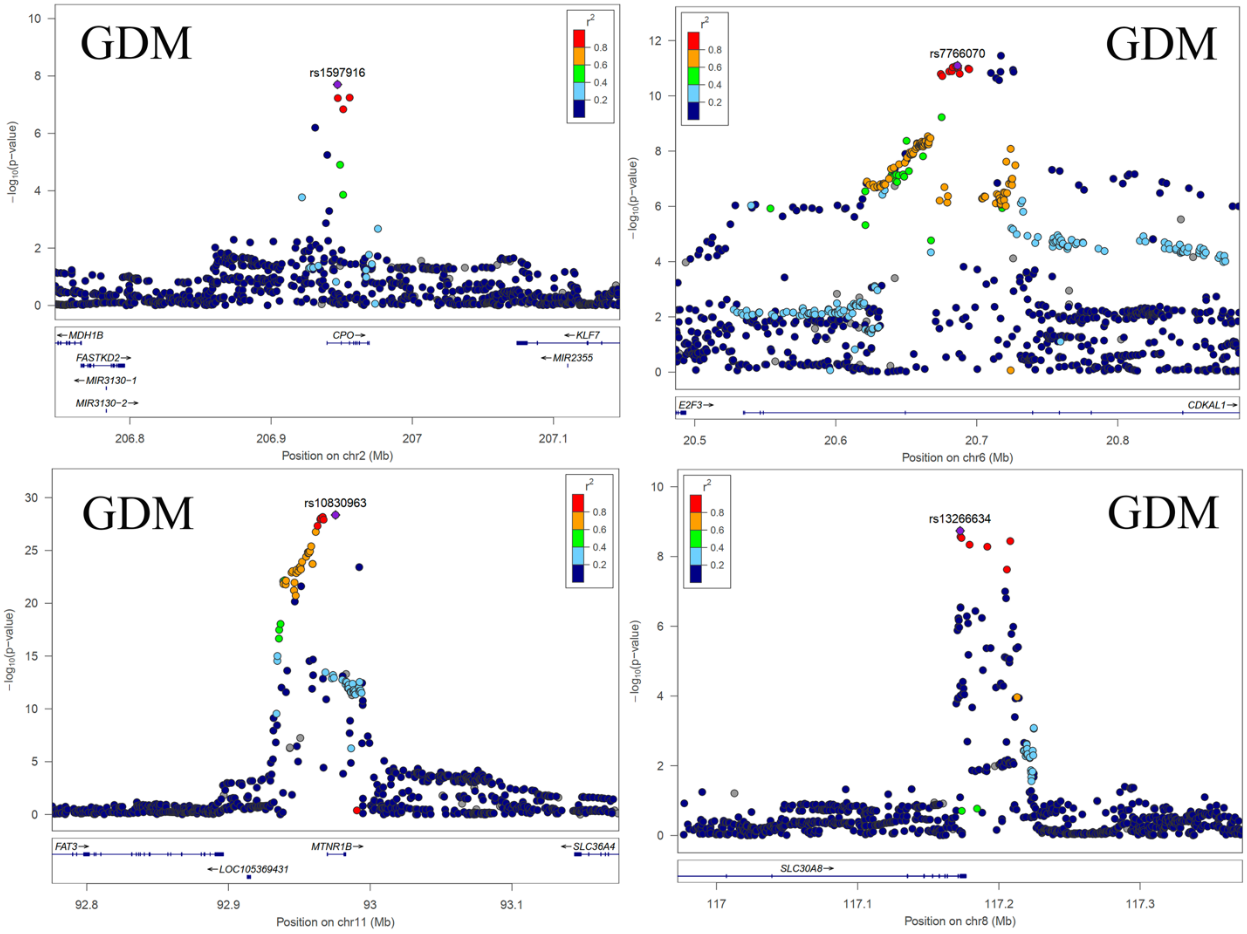
GDM
rs1597916
−log10(p−value)
r2
0.8
0.6
0.4
0.2
MDH1B
FASTKD2
MIR3130−1
MIR3130−2
CPO
KLF7
MIR2355
206.8
206.9
207
207.1
Position on chr2 (Mb)
GDM
rs7766070
E2F3
CDKAL1
20.5
20.6
20.7
20.8
Position on chr6 (Mb)
GDM
rs10830963
FAT3
LOC105369431
MTNR1B
SLC36A4
92.8
92.9
93
93.1
Position on chr11 (Mb)
GDM
rs13266634
SLC30A8
117
117.1
117.2
117.3
Position on chr8 (Mb)

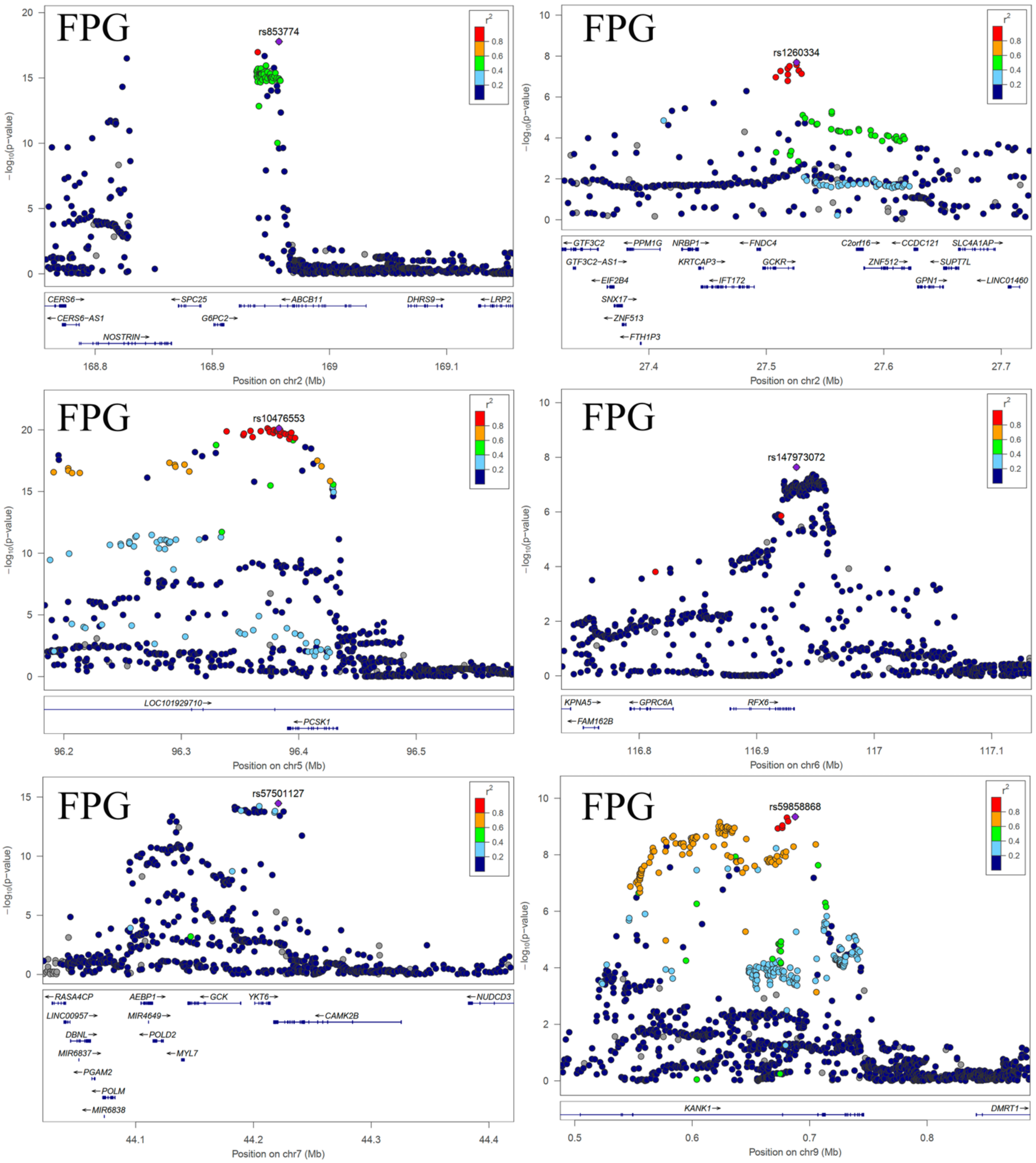
FPG
rs853774
CERS6→
←CERS6-AS1
NOSTRIN→
←SPC25
G6PC2→
←ABCB11
DHRS9→
←LRP2
Position on chr2 (Mb)
FPG
rs1260334
←GTF3C2
GTF3C2-AS1→
←EIF2B4
SNX17→
←ZNF513
←FTH1P3
←PPM1G
NRBP1→
KRTCAP3→
←IFT172
←FNDC4
GCKR→
C2orf16→
ZNF512→
←CCDC121
GPN1→
←SUPT7L
SLC4A1AP→
←LINC01460
Position on chr2 (Mb)
FPG
rs10476553
LOC101929710→
←PCSK1
Position on chr5 (Mb)
FPG
rs147973072
KPNA5→
←FAM162B
←GPRC6A
RFX6→
Position on chr6 (Mb)
FPG
rs57501127
←RASA4CP
LINC00957→
DBNL→
MIR6837→
←PGAM2
←POLM
←MIR6838
AEBP1→
MIR4649→
←POLD2
←MYL7
←GCK
YKT6→
←CAMK2B
←NUDCD3
Position on chr7 (Mb)
FPG
rs59858868
KANK1→
DMRT1→
Position on chr9 (Mb)
−log10(p-value)
r2

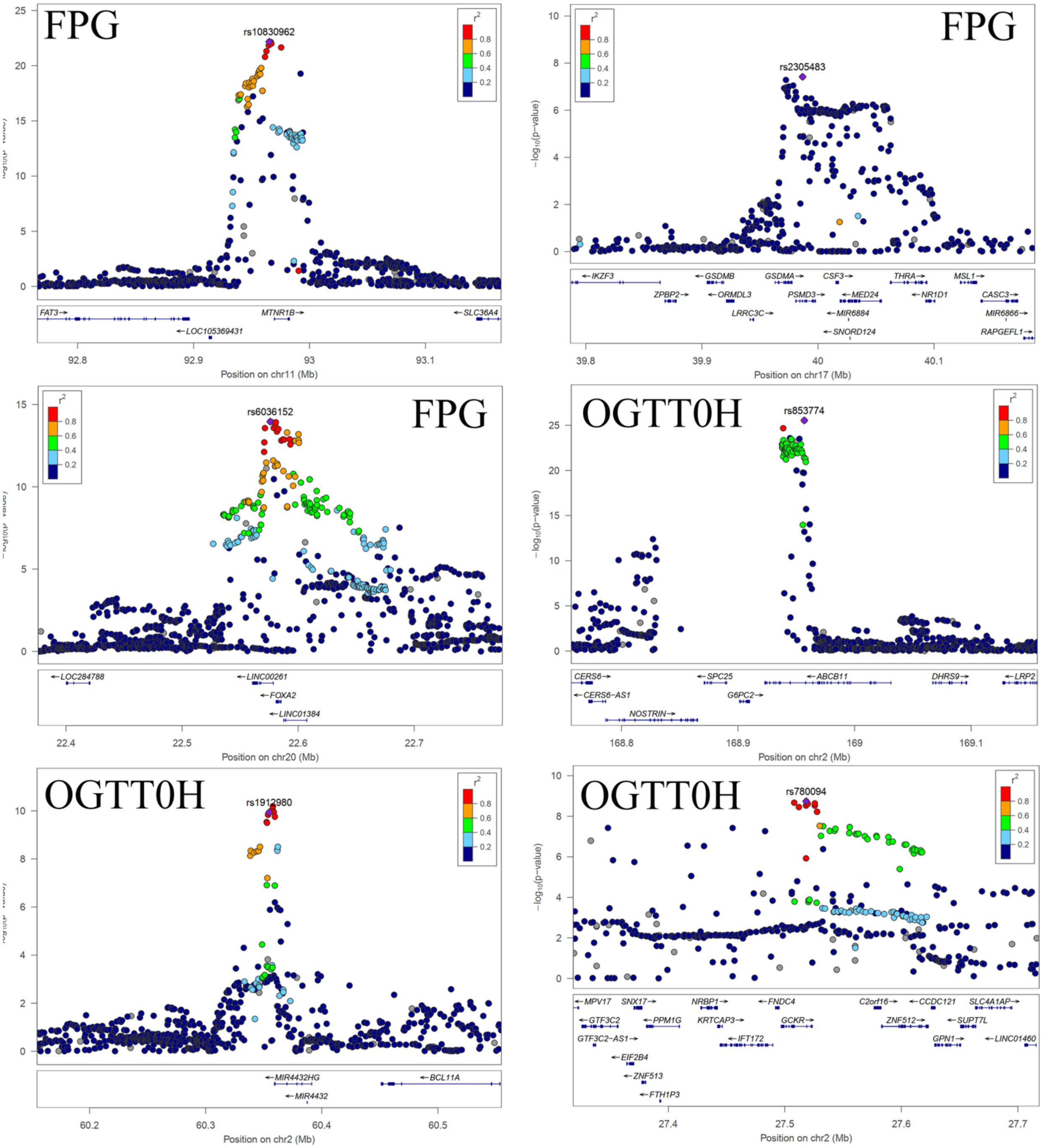
FPG
rs10830962
Position on chr11 (Mb)
FAT3
MTNR1B
SLC36A4
LOC105369431
FPG
rs2305483
Position on chr17 (Mb)
IKZF3
GSDMB
GSDMA
CSF3
THRA
MSL1
ZPBP2
ORMDL3
PSMD3
MED24
NR1D1
CASC3
LRRC3C
MIR6884
MIR6866
SNORD124
RAPGEFL1
FPG
rs6036152
Position on chr20 (Mb)
LOC284788
LINC00261
FOXA2
LINC01384
OGTT0H
rs853774
Position on chr2 (Mb)
CERS6
SPC25
ABCB11
DHRS9
LRP2
CERS6-AS1
G6PC2
NOSTRIN
OGTT0H
rs1912980
Position on chr2 (Mb)
MIR4432HG
BCL11A
MIR4432
OGTT0H
rs780094
Position on chr2 (Mb)
MPV17
SNX17
NRBP1
FNDC4
C2orf16
CCDC121
SLC4A1AP
GTF3C2
PPM1G
KRTCAP3
GCKR
ZNF512
SUPT7L
GTF3C2-AS1
IFT172
GPN1
LINC01460
EIF2B4
ZNF513
FTH1P3

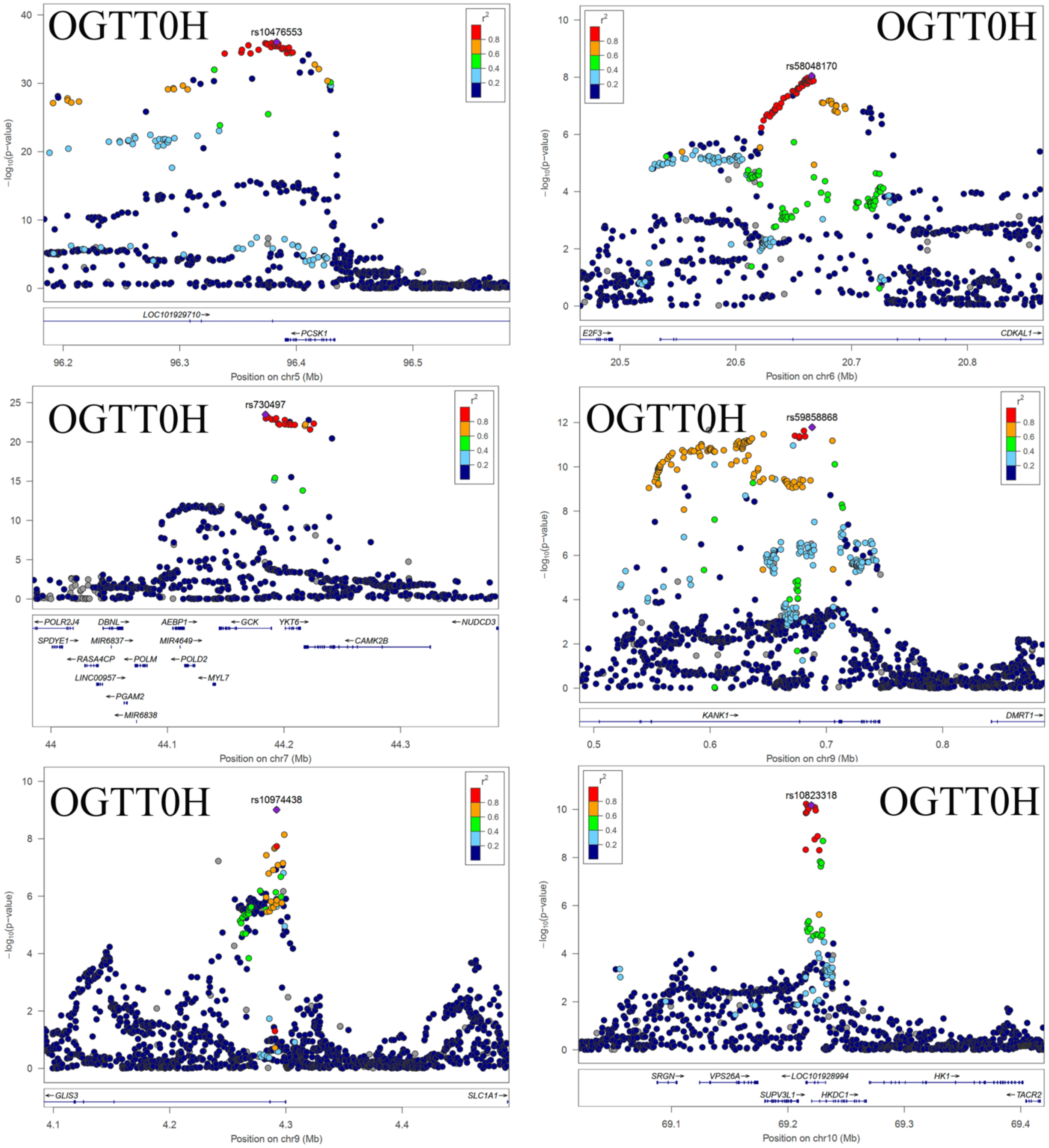

OGTT0H
rs10476553
LOC101929710
PCSK1
Position on chr5 (Mb)
OGTT0H
rs58048170
E2F3
CDKAL1
Position on chr6 (Mb)
OGTT0H
rs730497
POLR2J4
DBNL
AEBP1
GCK
YKT6
NUDCD3
SPDYE1
MIR6837
MIR4649
CAMK2B
RASA4CP
POLM
POLD2
LINC00957
MYL7
PGAM2
MIR6838
Position on chr7 (Mb)
OGTT0H
rs59858868
KANK1
DMRT1
Position on chr9 (Mb)
OGTT0H
rs10974438
GLIS3
SLC1A1
Position on chr9 (Mb)
OGTT0H
rs10823318
SRGN
VPS26A
LOC101928994
HK1
SUPV3L1
HKDC1
TACR2
Position on chr10 (Mb)
−log10(p−value)
r2

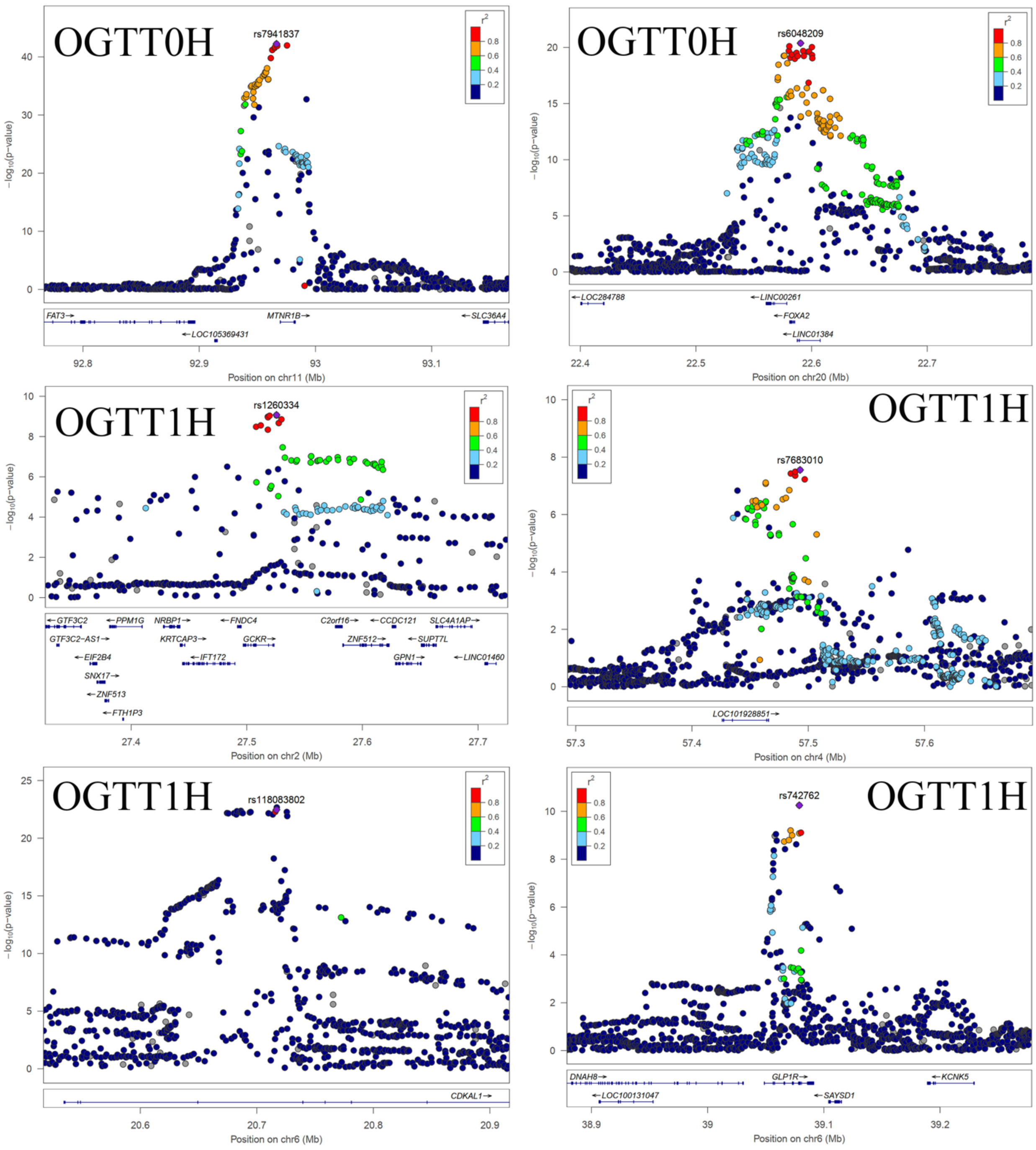
OGTT0H
rs7941837
FAT3→
←LOC105369431
MTNR1B→
←SLC36A4
Position on chr11 (Mb)
OGTT0H
rs6048209
←LOC284788
←LINC00261
←FOXA2
←LINC01384
Position on chr20 (Mb)
OGTT1H
rs1260334
←GTF3C2
←PPM1G
NRBP1→
←FNDC4
C2orf16→
←CCDC121
SLC4A1AP→
GTF3C2−AS1→
KRTCAP3→
GCKR→
ZNF512→
←SUPT7L
←EIF2B4
←IFT172
GPN1→
←LINC01460
SNX17→
←ZNF513
←FTH1P3
Position on chr2 (Mb)
OGTT1H
rs7683010
LOC101928851→
Position on chr4 (Mb)
OGTT1H
rs118083802
CDKAL1→
Position on chr6 (Mb)
OGTT1H
rs742762
DNAH8→
←LOC100131047
GLP1R→
←SAYSD1
←KCNK5
Position on chr6 (Mb)
−log10(p−value)
r2

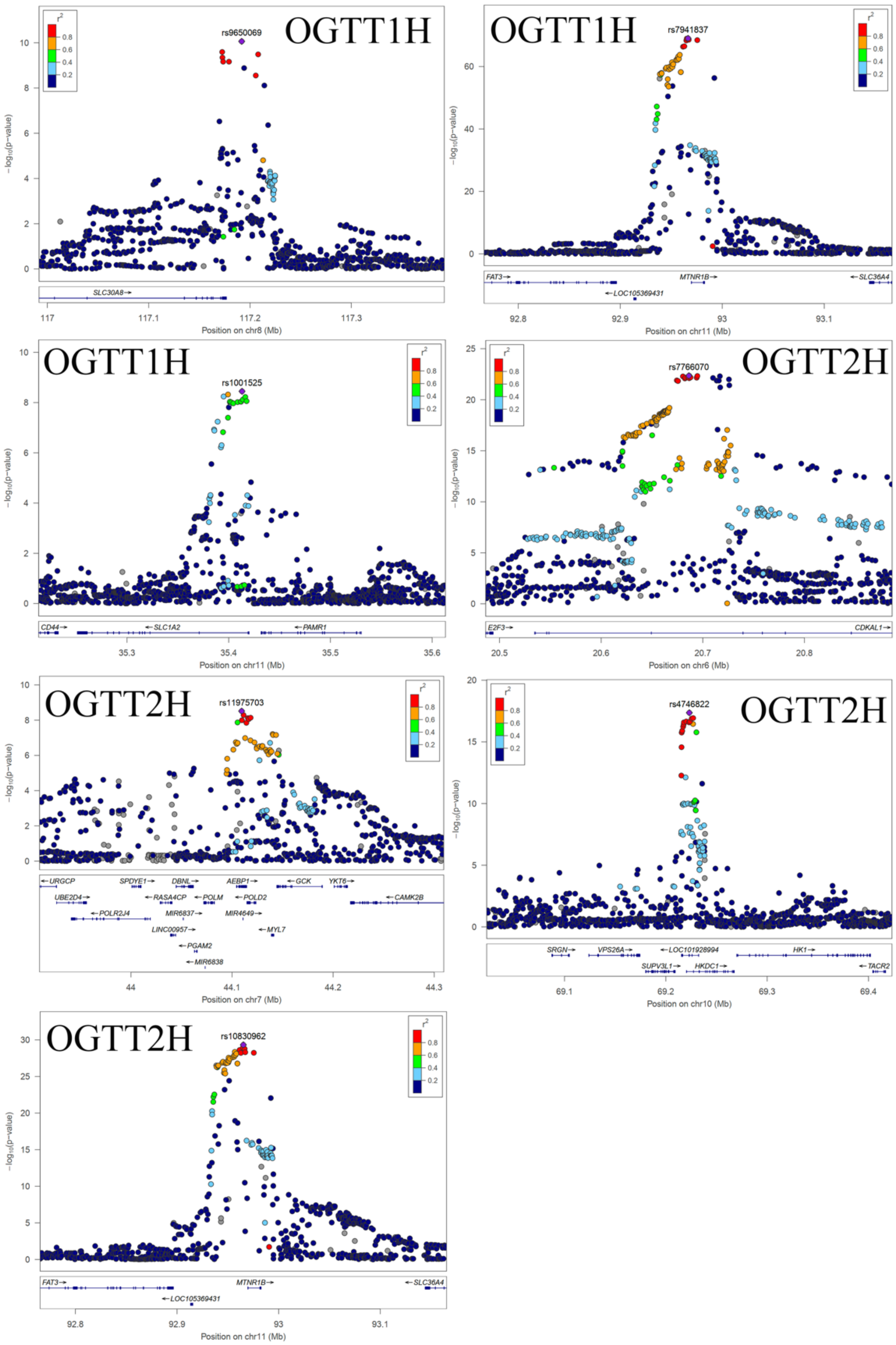

ESM Fig. 6 Locuszoom plot of genome-wide significant loci associated with the seven traits investigated in the study.

For all the 35 lead SNPs in ESM Table 8, a regional plot showing the P-value and the LD r^2 of SNPs located in the upstream and downstream 500kbp flanking region are demonstrated using the Locuszoom software.

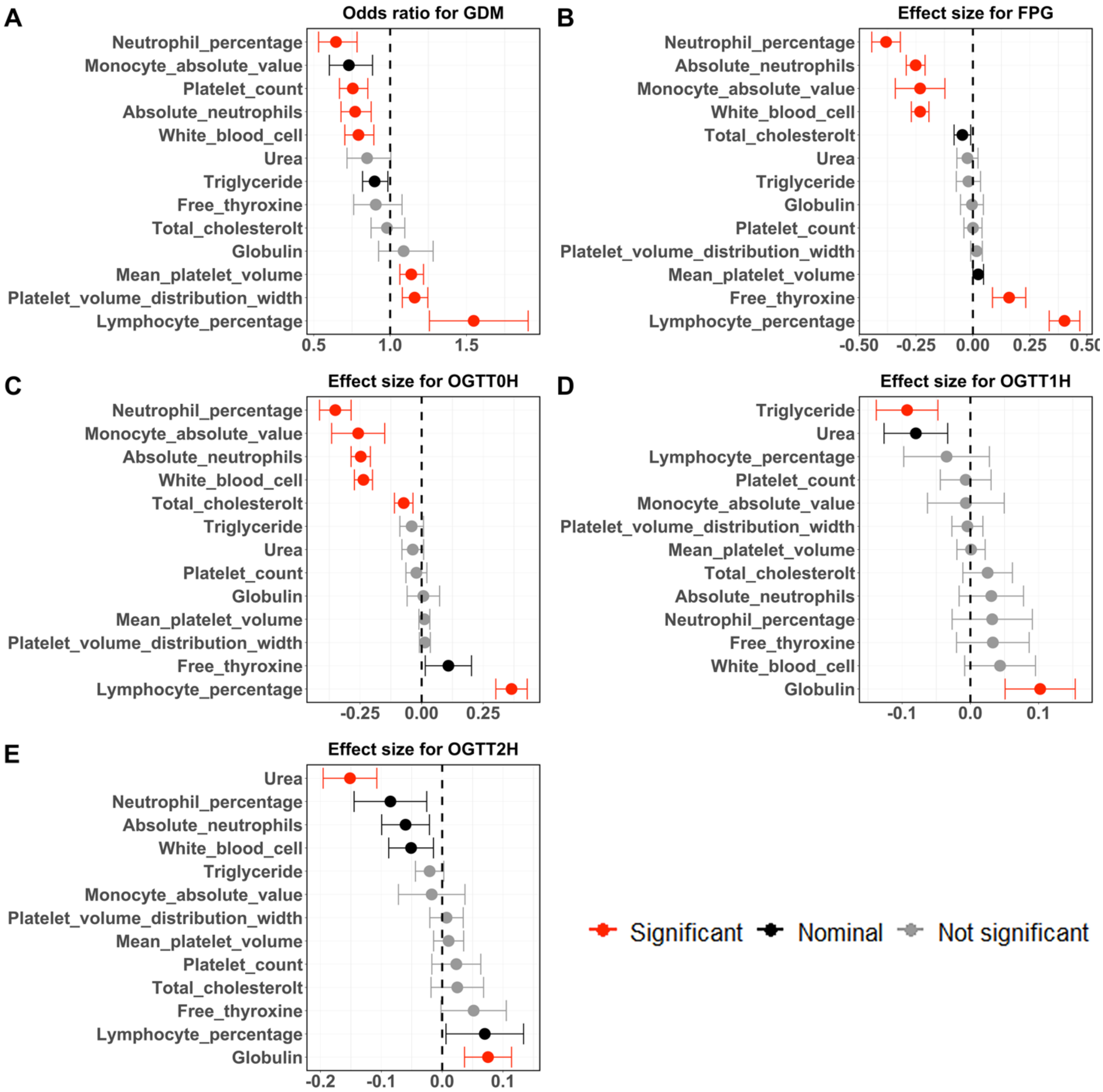

ESM Fig. 7 Effects of biomarkers on GDM and four glycemic traits by mendelian randomization.

All the 55 biomarkers collected in the pregnancy screening were analysed towards the GDM and the four quantitative glycemic traits.

Significant causal associations were marked in red where the following two criteria were met (1) P-values of inverse variance weight (IVW) was less than the bonferroni correction threshold (α =0.05/51/5) and (2) either the association showed no evidence of heterogeneity ($p > 0.05$) and horizontal pleiotropy ($p>0.05$), or one of the MR Egger, Simple mode, Weighted median, Weighted mode corrected estimates also have a $p<0.05$). Nominal significant causal association were marked in black using the similar criteria except that in criterion, P-value of IVW was less than 0.05 rather than less than bonferroni correction threshold. Associations that did not meet the significant or the nominal significant criteria were marked in gray.

Only biomarkers that demosntrate statistical significance after Bonferroni correction in at least one of the exposure-outcome analyses were included in the plot while results for all the biomarkers were presented in ESM Fig. 9.

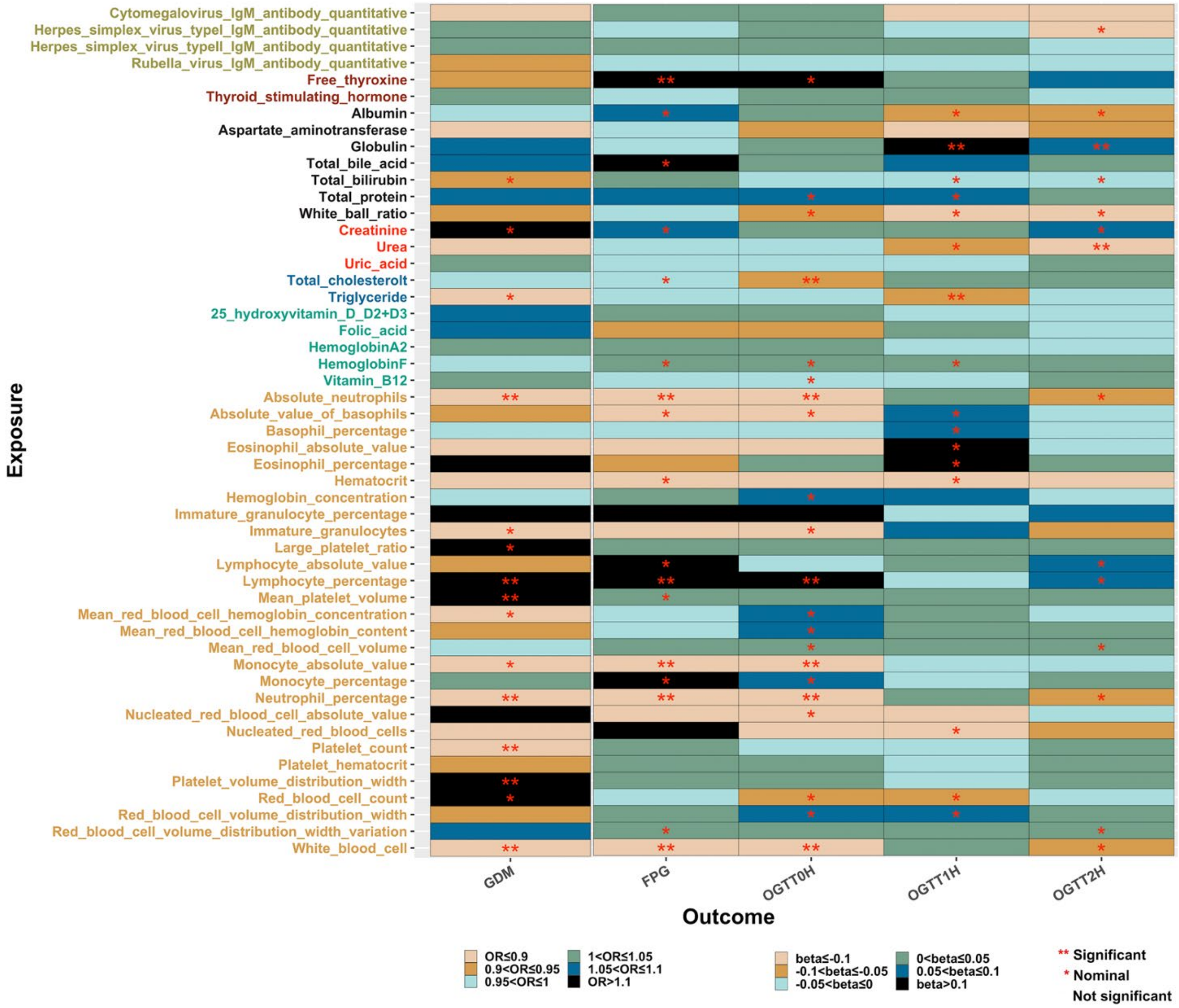

ESM Fig. 8 Effects of all 51 biomarkers on GDM and glycemic traits by mendelian randomization.

The rule for representing the color of the vertical coordinate, the color in the square was the same as for ESM Fig. 2. Square color indicate effect size of IVW, and the number of * sign in the square represents statistical significance, the rule of significance was the same as ESM Fig. 6, bonfferoni correction α=0.05/51/5.

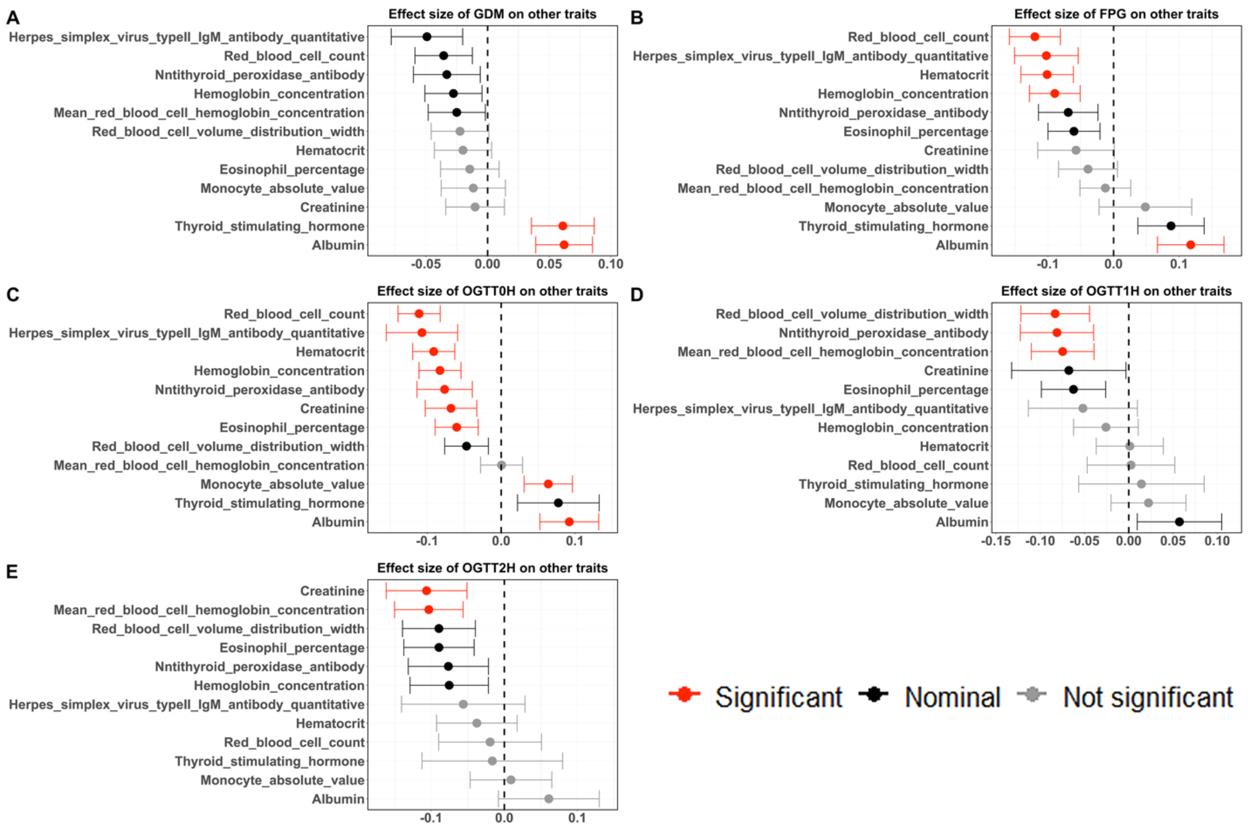

ESM Fig. 9 Effects of GDM and glycemic traits on the biomarkers by Mendelian randomization.

The analysis was conducted between all the 55 biomarkers collected in the pregnancy screening and the GDM occurrence as well as the 4 quantitative glycemic traits.

The definition of significance was the same as ESM Fig. 7, bonferroni correction α=0.05/55/5.

Only biomarkers that demosntrate statistical significance after Bonferroni correction in at least one of the exposure-outcome analyses were included in the plot while results for all the biomarkers were presented in ESM Fig. 11.

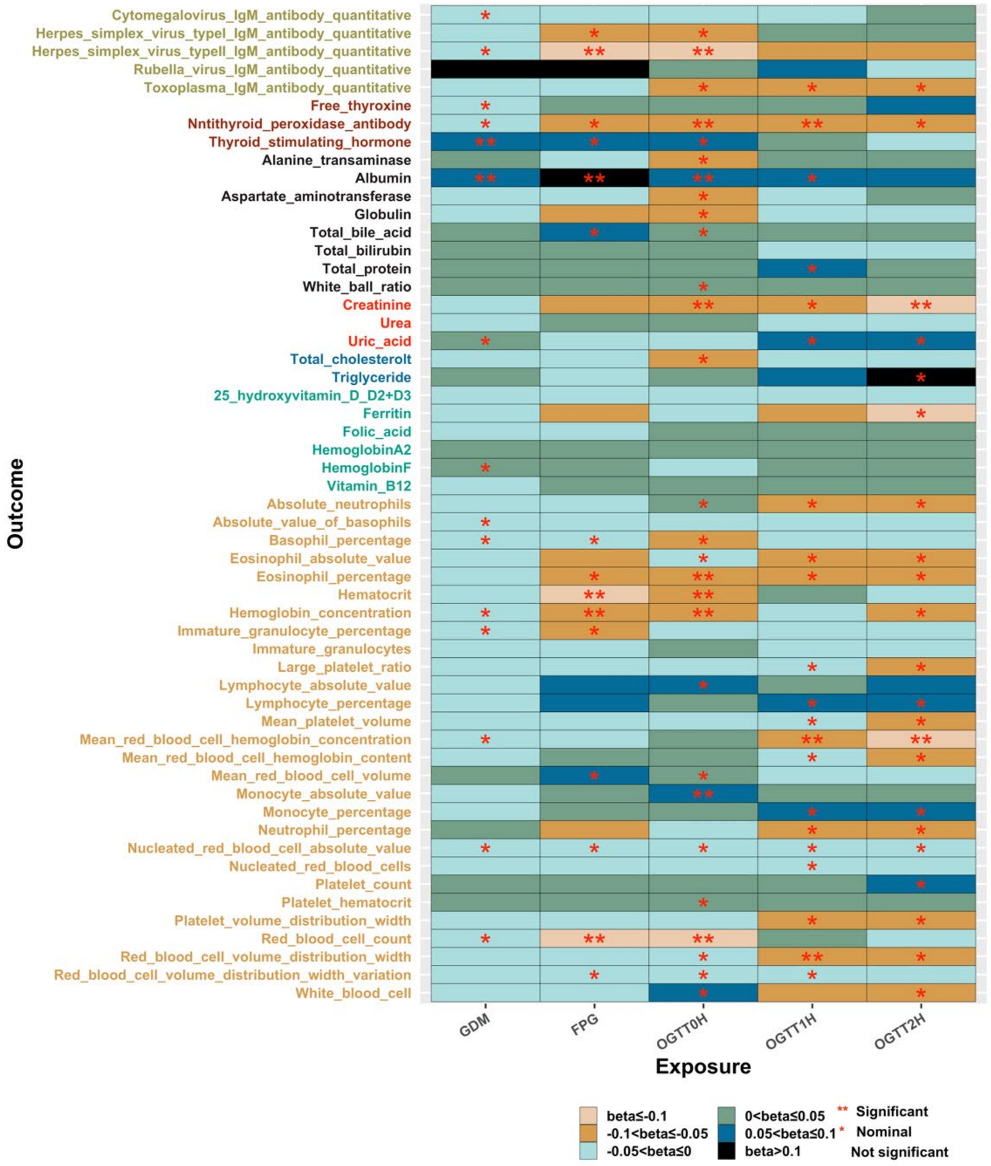

ESM Fig. 10 Effects of GDM and glycemic traits on all the 55 biomarkers by Mendelian randomization.

The rule for representing the color of the vertical coordinate, the color in the square was the same as for ESM Fig. 2. Square color indicate effect size of IVW, and the number of * sign in the square represents statistical significance, the definition of significance was the same as ESM Fig. 6, bonferroni correction α=0.05/55/5.

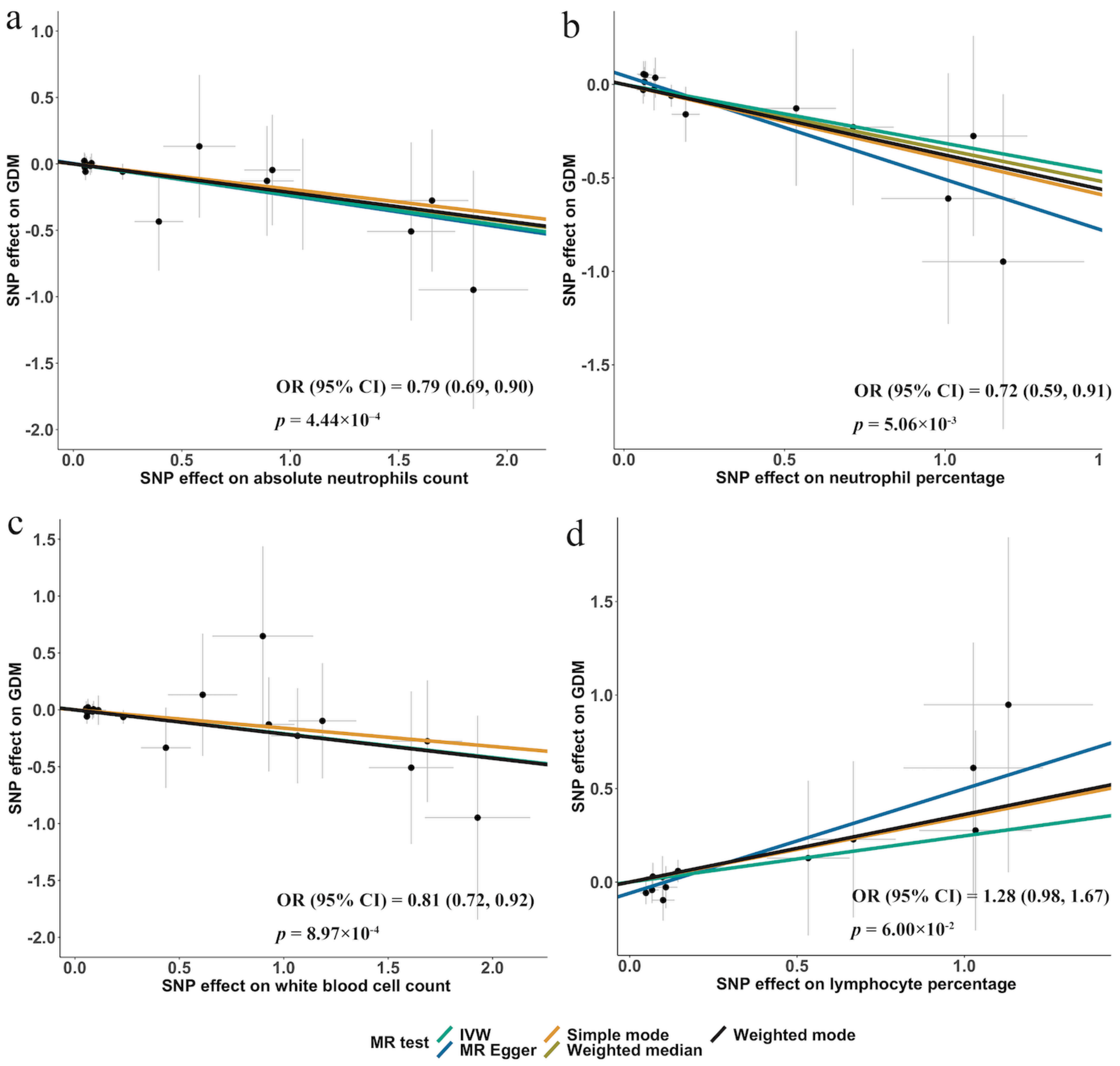

ESM Fig. 11 Scatter plot of mendelian randomization analysis results with 4 biomarkers as the exposure and their effect on GDM with PLUS cohort meta data.

Panel a-d presents the results of the mendelian randomization analyses using absolute neutrophils, neutrophil percentage, white blood cell and lymphocyte percentage as exposure respectively. The effect size and P value of inverse variance weighted showed in figure and the slope of the regression line in each panel indicates the direction of the effect of the exposure on GDM, with a positive slope representing a positive effect and a negative slope indicating a negative effect.
